# Benchmarking differential abundance methods for finding condition-specific prototypical cells in multi-sample single-cell datasets

**DOI:** 10.1186/s13059-023-03143-0

**Published:** 2024-01-03

**Authors:** Haidong Yi, Alec Plotkin, Natalie Stanley

**Affiliations:** 1 Department of Computer Science, University of North Carolina at Chapel Hill, 27599 Chapel Hill, NC USA; 2 Curriculum in Bioinformatics and Computational Biology, University of North Carolina at Chapel Hill, 27599 Chapel Hill, NC USA; 3 Computational Medicine Program, University of North Carolina at Chapel Hill, 27599 Chapel Hill, NC USA

**Keywords:** Benchmarking, Differential abundance (DA), Clinical phenotyping, Single-cell bioinformatics

## Abstract

**Background:**

To analyze the large volume of data generated by single-cell technologies and to identify cellular correlates of particular clinical or experimental outcomes, differential abundance analyses are often applied. These algorithms identify subgroups of cells whose abundances change significantly in response to disease progression, or to an experimental perturbation. Despite the effectiveness of differential abundance analyses in identifying critical cell-states, there is currently no systematic benchmarking study to compare their applicability, usefulness, and accuracy in practice across single-cell modalities.

**Results:**

Here, we perform a comprehensive benchmarking study to objectively evaluate and compare the benefits and potential downsides of current state-of-the-art differential abundance testing methods. We benchmarked six single-cell testing methods on several practical tasks, using both synthetic and real single-cell datasets. The tasks evaluated include effectiveness in identifying true differentially abundant subpopulations, accuracy in the adequate handling of batch effects, runtime efficiency, and hyperparameter usability and robustness. Based on various evaluation results, this paper gives dataset-specific suggestions for the practical use of differential abundance testing approaches.

**Conclusions:**

Based on our benchmarking study, we provide a set of recommendations for the optimal usage of single-cell DA testing methods in practice, particularly with respect to factors such as the presence of technical noise (for example batch effects), dataset size, and hyperparameter sensitivity.

**Supplementary Information:**

The online version contains supplementary material available at 10.1186/s13059-023-03143-0.

## Background

**Fig. 1:**
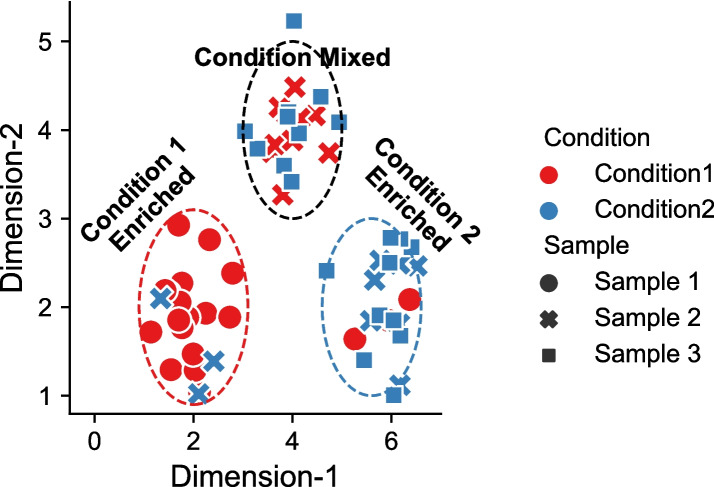
An illustration of DA effects in a toy single-cell dataset containing three samples under two experimental conditions. Cells from condition 1 are enriched in lower left region (red circle), while cells from condition 2 is enriched in lower right region (blue circle). The upper region (black circle) is equally mixed with cells from both the condition 1 and 2 without DA effects

In studying data generated by modern single-cell technologies, differential abundance (DA) testing methods [1–6] have become indispensable for uncovering cellular correlates of particular biological, developmental, and disease states [7]. Inspired by early developments in microbiome-based DA analysis [8–12], these methods broadly highlight significant differences in cellular compositions between conditions (see Fig. 1 for an intuitive illustration). Despite the challenges posed by the inherent heterogeneity in single-cell data, DA analysis techniques, including both clustering-based and clustering-free variants, offer insights into phenotype or outcome-associated cell subsets. Clustering-based methods, such as Diffcyt and Cydar [1, 2], are highly dependent on having appropriately binned cells into phenotypically coherent cell populations, which may pose limitations in identifying novel, and functionally distinct cellular subsets. In contrast, clustering-free strategies, such as Meld, Milo, Cna, and DA-seq introduced in Refs. [3–6], excel at inferring condition-specific per-cell importances, which offers a more granular approach for identifying outcome-associated cells.

Differential abundance approaches have be productively used in practice in both clinical and experimental settings. For example, these approaches highlighted an increased presence of granulocytes, monocytes, and B cells in fatal cases of COVID-19 [13]. They also led to the discovery of cell populations associated with rheumatoid arthritis [6] and the unveiling of a novel subset of cells in mouse intertropical thymic epithelial cells (TECs) that diminish with age [5]. Despite the fact that numerous research efforts focused on developing new DA methods, there have been considerably few studies providing thorough and quantitative comparisons of the strengths and weaknesses of the common DA testing approaches, such as Milo [5], Meld [4], and Cna [6]. Currently, across original papers introducing differential abundance methods, Milo [5] is the only work to provide a quantitative evaluation of aspects of accuracy across methods. However, further work is needed to systematically benchmark and understand dataset or modality-specific trends in interpretability in usability in extracting biological insights across DA testing methods. To bolster the quantitative and objective comparison between methods at scale, we designed the first comprehensive benchmarking study to compare several nuances of the current state-of-the-art differential abundance testing methods.

Specifically, in this benchmarking study, we evaluated six DA testing methods proposed for single-cell omics data analysis. The methods span both clustering-based and cluster-free approaches, and we systematically applied these methods across both synthetic and real single-cell datasets. We examined various aspects of the usability of the DA methods in practice, including, (1) the precision in detecting DA subpopulations in data with diverse differential trajectory structures; (2) the capacity to handle technical and biological variables, such as batch effects; (3) runtime efficiency and scalability; and (4) usability and robustness with regard to hyperparameters. To summarize a key insight gleaned across all experiments, we reasoned that several DA methods cannot perform well when the number of cells is significantly unbalanced between DA subpopulations, which is a likely phenomenon to arise in practice. After investigating the characteristics of each method in relation to the unique characteristics of our diverse datasets studied, we ultimately provided data-specific suggestions for choosing the best DA approaches to use in particular settings.

## Results

### Benchmarking overview

In this benchmarking study, we objectively and quantitatively compared current state-of-the-art DA testing methods in their capacity to accurately identify cell-populations that were associated with clinical or experimental outcomes of interest. To do so, we examined six prevalent DA approaches and evaluated them on three synthetic and four real single-cell datasets [7, 14–16]. To facilitate a thorough benchmarking study, the experimental datasets differ in several ways and include variations in cell counts, topology of differential trajectories, ratio or magnitude of the differential abundance effect (DA ratio), and single-cell modality (e.g., protein vs. gene measurement). The three synthetic datasets, for example, have different topological structures (linear, branch, and cluster) of their differential trajectories and DA ratios (see Additional file 1: Fig. S1). The real datasets are comprised of two single-cell RNA sequencing (scRNA-seq) datasets–COVID-19 PBMC [7] and Human Pancreas [16]–and two mass cytometry (CyTOF) datasets-BCR-XL [14] and Levine32 [15] with manually annotated cell-type labels (see Additional file 1: Fig. S2). To evaluate scalability of these approaches, we also simulated six additional scRNA-seq datasets, with cell counts ranging from 4000 to 100,000.

**Fig. 2:**
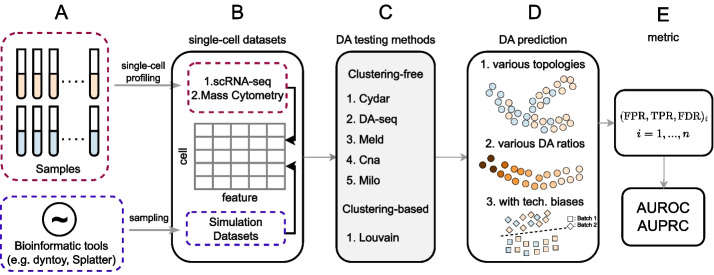
Schematic illustration of the benchmarking workflow. Using both synthetic and real single-cell datasets, six DA testing methods were evaluated under three configurations for the DA prediction task. **A**, **B **Single-cell RNA-seq and mass cytometry datasets were generated from patient samples, or synthetic datasets were generated using the packages dyntoy [20] or splatter [18]. **C**, **D **Next, we evaluated the six clustering-based [17] and clustering-free [1, 3–6] DA testing methods on datasets with different topologies, DA ratios, and technical biases such as batch effects. **E** Lastly, we compare the performance of the DA testing methods using both the AUROC and AUPRC scores

Figure 2 depicts our benchmarking workflow. We evaluated a total of six distinct DA testing approaches, which can be broadly categorized into two groups: (i) clustering-based methods [17] and (ii) clustering-free methods [1, 3–6]. For clustering-free methods, we benchmarked five methods including Cydar [1], Milo [5], DA-seq [3], Meld [4], and Cna [6]. Noting that clustering-free methods often exhibit superior performance in comparison to clustering-based methods, we compared all such results to those attained through the Louvain algorithm [17], a commonly used graph-based clustering method in single-cell data analysis. To further show the differences of the six DA testing methods, we compare their properties in Additional file 1: Table S1. Here, we provide a brief summary of the six approaches included in this benchmarking study. For more implementation details, see the “Differential abundance (DA) testing methods” section in the “Methods” section.

1. Cydar [1]: Cydar detects DA cell populations by assigning cells to hyperspheres and testing whether the number of cells in each hypersphere varies in a statistically significant way between conditions. The spatial false discovery rate (FDR) throughout the high-dimensional space controls Cydar’s type I error
2. DA-seq [3]: DA-seq predicts DA scores for each cell under two separate conditions by applying a logistic regression model. Label permutation is then used to empirically evaluate the statistical significance of the prediction results
3. Meld [4]: Meld calculates the likelihood that each cell belongs to or is prototypical of each condition, using a graph-based kernel density estimation (KDE) method. The DA cells are then selected by setting a heuristic likelihood threshold
4. Cna [6]: Cna uses random walks on graphs to generate a neighborhood abundance matrix (NAM), which quantifies the relative abundance of each sample within particular cellular neighborhoods. DA cell-populations are then ultimately identified through statistical testing based on the NAM across the conditions
5. Milo [5]: Milo begins by counting the number of cells from each sample within *k*-nearest neighborhoods and then applies a negative binomial generalized linear models (NB-GLM) to test the DA of each local graph (first order neighborhood). Milo, like Cydar, controls type-I error via spatial FDR
6. Louvain [17]: The Louvain method first clusters cells across samples using the Louvain algorithm and then counts the cells of each sample within each cluster. Louvain further uses the same procedure as Milo to ultimately determine the DA cells

Due to the fact that the six methods employ diverse strategies to estimate DA cell populations, we had to design a principled way to compare between these varied approaches. To do so, we used the area under the receiver operator curve (AUROC) and precision-recall curve (AUPRC) scores to quantify the accuracy of these approaches in practice. Noting that our datasets do not have ground truth DA labels, we employed a data-driven technique to construct such labels for each individual cell after setting the target DA cell populations (see the “Generation of ground-truth DA labels” section in the “Methods” section). By aggregating results across all datasets with different experimental configurations, we ultimately ranked the overall predictive performance of the six DA testing methods. This gauges how well cell populations that are strongly related with the corresponding conditions, such as phenotype and experimental perturbations, can be inferred via DA testing.

In addition to accurately inferring the condition-specific cells, further issues should be addressed to make DA testing methods more applicable in real-world settings. First, in order to provide reliable predictions, a DA testing method must be robust to other variables in a dataset, such as batch effects and sample covariates. Second, a DA testing method should be resilient to datasets with different characteristics. For instance, (1) there are numerous single-cell profiling modalities, such as scRNA-seq and CyTOF, which measure the expression of genes and proteins, respectively; (2) the structure of differential trajectories in single-cell datasets varies in high-dimensional gene or protein expression space; and (3) the size and differential abundance ratio of DA cell populations can vary between datasets. Finally, a practical DA testing method should be computationally efficient, such that it can be readily applied to single-cell datasets containing more than 100,000 cells. Hence, we ran a series of experiments to evaluate and compare the performance of DA testing methods in various configurations and to see if they are capable of handling the challenges above. Furthermore, we also carried out studies to examine the sensitivity of results with respect to the input hyperparameters. This is crucial because, in practical applications, it might be difficult for users to specify an appropriate hyperparameter as input for a new given dataset without some background knowledge (see the “Hyperparameter tuning and sensitivity” section). In our implementation, the hyperparameters were tuned as suggested in the original work. For specific hyperparameter values used in our experiments, refer to Additional file 1: Table S2.

### DA testing performance on synthetic datasets

**Fig. 3:**
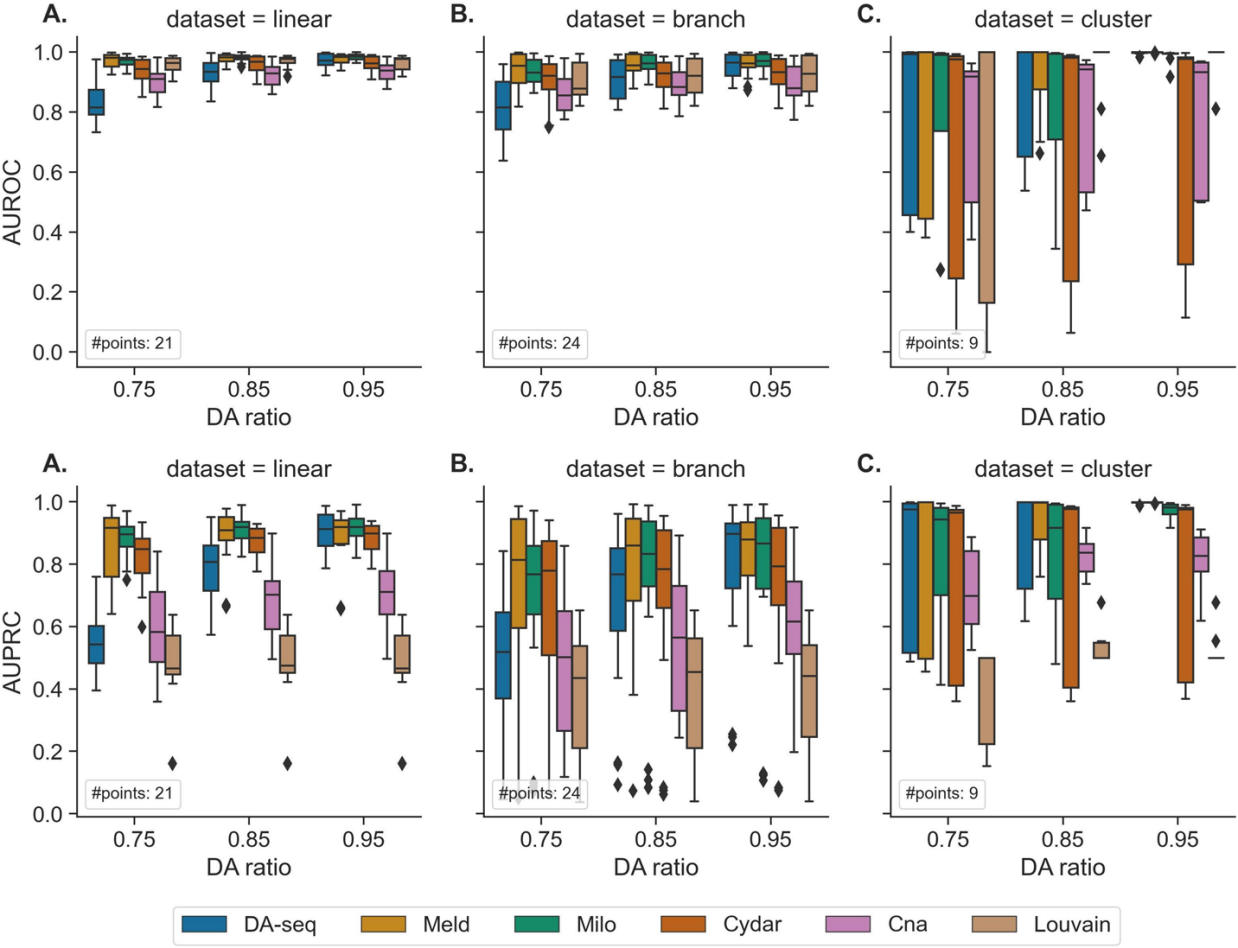
Performance of the six DA testing methods for DA prediction on the three synthetic datasets (linear (**A**), branch (**B**), and cluster (**C**)) with a range of DA ratios (0.75, 0.85, and 0.95) in the target DA cell population. The boxplots represent the distributions of AUROC and AUPRC scores for various target DA cell populations across multiple random seeds. #points represents the number of points for each boxplot

First, the DA testing methods were evaluated on three synthetic datasets, referred to throughout the text as *linear*, *branch*, and *cluster* (see Additional file 1: Fig. S1). Figure 3 and Additional file 1: Tables S3  and S4 show the performance of the six DA testing methods on the three synthetic datasets and generally show how the performance changes with respect to DA ratios. On the synthetic datasets, we represented each individual high-dimensional cell in terms of its top 50 principal components (PCs) and systematically evaluated performance across varying DA ratios, target cell populations, and random seeds. When comparing the performance of the DA testing methods, we unsurprisingly observed that the accuracy of all DA methods as evaluated with AUROC and AUPRC increased consistently as the DA ratio increased from 0.75 to 0.95 across the three datasets. This suggests that a higher DA ratio leads to a simpler and less-noisy DA testing problem (Fig. 3). To quantify the performance of each method, we evaluated both AUROC and AUPRC scores, averaging the median results across all DA ratios. The average AUROC scores for DA-seq, Meld, Milo, Cydar, Cna, and Louvain on the linear dataset were 0.91, 0.98, 0.98, 0.96, 0.93, and 0.97, respectively, while their respective AUPRC scores were 0.75, 0.92, 0.91, 0.88, 0.67, and 0.47 (Additional file 1: Tables S3 and S4). On the linear dataset, when considering the AUROC metric, all the six methods are very effective for predicting DA cell populations. When examining the AUPRC metric, it is notable that Meld, Milo, and Cydar maintained their scores around 0.9, while other methods dropped their performance significantly. DA-seq demonstrated AUPRC scores below Meld, Milo, and Cydar but superior to Cna and Louvain. Similar patterns were also observed in the branch dataset (Fig. 3B). The average AUROC scores for DA-seq, Meld, Milo, Cydar, Cna, and Louvain on the branch dataset were 0.90, 0.96, 0.96, 0.93, 0.87, and 0.91, respectively, while their respective AUPRC scores were 0.73, 0.85, 0.83, 0.79, 0.56, and 0.44 (Additional file 1: Tables S3 and S4). Noting that the DA testing methods provided similar performance and relative performance rankings on the linear and branching datasets, we hypothesize that this was due to their similar differential trajectories.

On the cluster dataset, the averaged AUROC scores for DA-seq, Meld, Milo, Cydar, Cna, and Louvain were 1.00, 1.00, 1.00, 0.98, 0.93, and 1.00, respectively, while the corresponding AUPRC scores were 0.99, 1.00, 0.95, 0.97, 0.79, and 0.50. (Fig. 3C). Since the cluster dataset has a simpler topology, the performance of all the methods was better than their results on the linear and branch datasets. When examining the distribution of AUROC scores across various target DA cell populations, we found extremely high variance, especially for DA ratios of 0.75 or 0.85. In addition, the imbalanced distribution of AUROCs (high median value and variance) in Fig. 3C revealed that the DA testing methods performed very poorly in a small subset of the experiments. To further explore this, we visualized boxplots of AUROC scores for each target DA cell population on the cluster dataset (Additional file 1: Fig. S3). This suggested that the DA testing methods perform well and consistently when the target DA population is M1 cell type or M3 cell type, whereas for the M2 population (Additional file 1: Fig. S3A), all the methods had a significant drop in performance compared to M1 and M3. To understand this, we checked the predictions and found that the DA testing methods generate more false negative results when M2 is the target DA population. Since the M2 population contained significantly more cells than the M1 and M3 populations, we hypothesized that this is due to the heterogeneity of DA effects in the M2 populations.

To validate our hypothesis, we implemented a subsampling technique to reduce the M2 population and subsequently conducted another experiment using a balanced cluster dataset. When the target DA population was M2 on the balanced cluster dataset, the performance of each method was greatly improved (Additional file 1: Fig. S3B). Further comparisons with results from the initial cluster dataset (Additional file 1: Fig. S3A) revealed a notable reduction in the variance of AUROC scores across the M1, M2, and M3 target populations within the balanced dataset. Our experiments thereby revealed that the DA testing methods tend to prioritize local differential abundance. If faced with a large DA cell population exhibiting heterogeneous DA effects, these methods might struggle with accurately determining the entire cell population as differentially abundant.

### DA testing performance on scRNA-seq and CyTOF Datasets

**Fig. 4:**
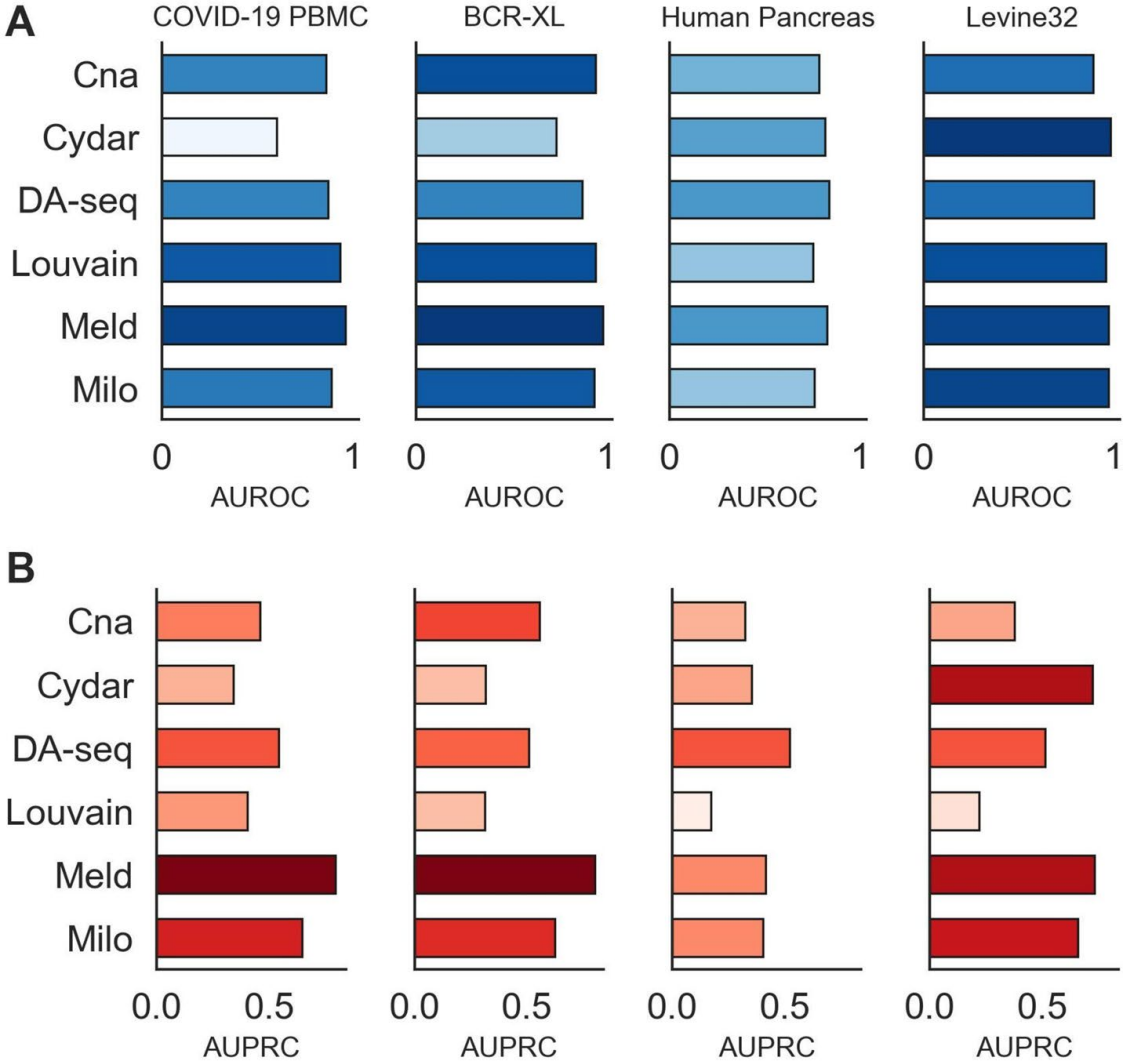
Performance of the six DA testing methods for DA prediction on the four real single-cell datasets with a range of DA ratios (0.75, 0.85, and 0.95) across the target DA cell types. The bar plots represent the AUROC (**A**) and AUPRC scores (**B**) for different target DA cell types evaluated over different random seeds

Next, we used two scRNA-seq (COVID-19 PBMC and Human Pancreas) and two CyTOF (BCR-XL and Levine32) datasets to benchmark DA testing methods in real single-cell datasets. Consistent with the patterns observed in the synthetic datasets, we also observed that the performance of all methods improved steadily as the DA ratio increased (Additional file 1: Tables S5  and S6). Similarly, the variance of AUROC scores also decreased as the DA ratio increased across datasets for all methods except for Cydar. This showed that, similar to the patterns observed in the synthetic dataset, the DA ratio can significantly affect the performance and stability of the DA testing methods. The averaged AUROC and AUPRC scores across the six DA testing methods and real datasets were shown in Fig. 4 and Additional file 1: Tables S5 and S6. For AUROC scores, all the methods achieved strong overall performances and Meld was ranked first across the datasets, followed by Milo, Louvain, DA-seq, Cna, and Cydar. Milo and Louvain performed similarly, and DA-seq and Cna also performed similarly. For AUPRC scores, Meld was ranked first among the six DA testing methods and across the real datasets as well, surpassing Milo, DA-seq, Cna, Cydar, and Louvain. Overall, the methods’ performances and rankings remained consistent in both the synthetic and real single-cell datasets, demonstrating that their performances are independent of the data but are reflections of their own capabilities. For example, the performance of Meld and Milo were robust and both ranked top-2 on the synthetic and real datasets. In addition, all the methods showed a significant decrease in AUPRC scores on the real datasets, indicating that DA testing task is more difficult for real datasets. We also noticed that Cydar showed a considerable decrease in its AUROC score on the real datasets. This demonstrated that Cydar is not robust to the biases between synthetic and real datasets, or it may have been sensitive to changes in other factors, such as hyperparameters.

### DA testing performance on datasets with additional technical and biological covariates

**Fig. 5:**
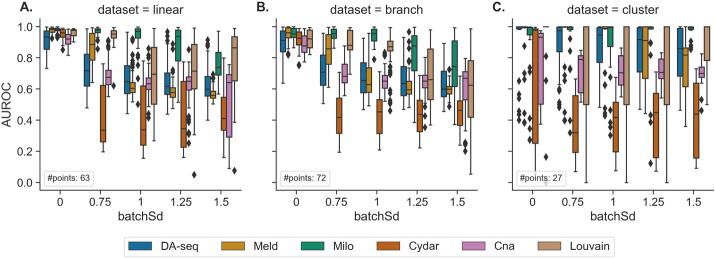
Performance of the six DA testing methods in identifying differentially abundant cell-types in three synthetic datasets (linear (left), branch (middle), and cluster (right)) with batch effects of varying magnitudes (from 0 to 1.5). When batchSd=0, no batch effects are present. The boxplots represent the AUROC scores for different target DA cell populations, DA ratios, and random seeds. #points represents the number of points for each boxplot

In addition to the clinical outcomes used in DA testing, such as clinical phenotype or disease status, single-cell datasets are often affected by additional technical and biological factors, such as batch effects, donor type, and cell cycle artifacts. These undesirable variables provide additional variance in the data and can confound the biological variations in the subsequent analysis, resulting in more false positives. In this subsection, we examine how the performance of DA testing methods changes when batch effects are present in the data, as well as how each DA testing method particularly accounts for additional covariates, such as batch effects. Out of the six DA testing methods, Cydar, Milo, Cna, and Louvain can explicitly include such external variables into their testing models to account for variance, whereas DA-seq and Meld do not. Following the procedure in Ref. [5], we simulated batch effects with a variety of magnitudes and added them to the three synthetic datasets. We then evaluated the performance of the six DA testing methods on the synthetic datasets containing batch effects (Fig. 5). To exclude impact from different hyperparameters, we used the same hyperparameter values that were used when no batch effects were present. Overall, the results demonstrated that all DA methods exhibited poorer performance when batch effects were present in the data, in comparison to batch-effect free data, as exhibited by a decline in AUROC scores with increasing magnitude of batch effects (Fig. 5). The imbalance of class labels in our prediction task (Fig. 5) potentially skewed classification thresholds, which further worsened the performance of some methods (e.g., Cydar) with AUROC scores < 0.5. This showed that technical artifacts such as batch effects had a significant adverse effect on the quality of DA testing methods. Of the six DA testing methods, Milo consistently performed the best over a range of batch effect magnitudes. Despite no explicit implementation to include batch labels in their models, DA-seq and Meld were inferior to the performance of Milo but comparable to other methods like Cna and Louvain. We also noticed a strong negative link between how well DA-seq and Meld worked and how strong the batch effects were (Fig. 5). Cydar, Cna, and Louvain were the weakest methods for handling batch effects as their performances were affected by even slight batch effects. Moreover, in Fig. 5A, Louvain and Cydar did not exhibit significantly improved performance when decreasing batch effect magnitude, suggesting a varying sensitivity to batch effects among different methods. Since we used the same hyperparameters across orders of batch effect magnitude in Fig. 5 for each method, this unexpected trend for Cydar and Louvain can also be attributed to their high sensitivity to hyperparameter choice. Moreover, this observation is consistent with our results in “Hyperparameter tuning and sensitivity” section.

In addition, we conducted a second experiment to examine whether incorporating batch labels into the models could improve the performance. In this experiment, we applied Cydar, Milo, Cna, and Louvain on synthetic datasets using two different setups. In the first setup, batch labels were included in the models, whereas in the second setup, they were omitted. Additional file 1: Fig. S5 illustrated the performance of these four approaches with or without inclusion of batch information. We discovered that, with the exception of Cydar, explicitly modeling batch effects can greatly enhance the performance of DA testing procedures in datasets with prominent batch effects. Furthermore, these experiments demonstrated that it is crucial for DA testing procedures to account for the variance introduced by additional technical and biological factors in order to produce accurate and meaningful results.

### Runtime efficiency and scalability of DA testing methods

**Fig. 6:**
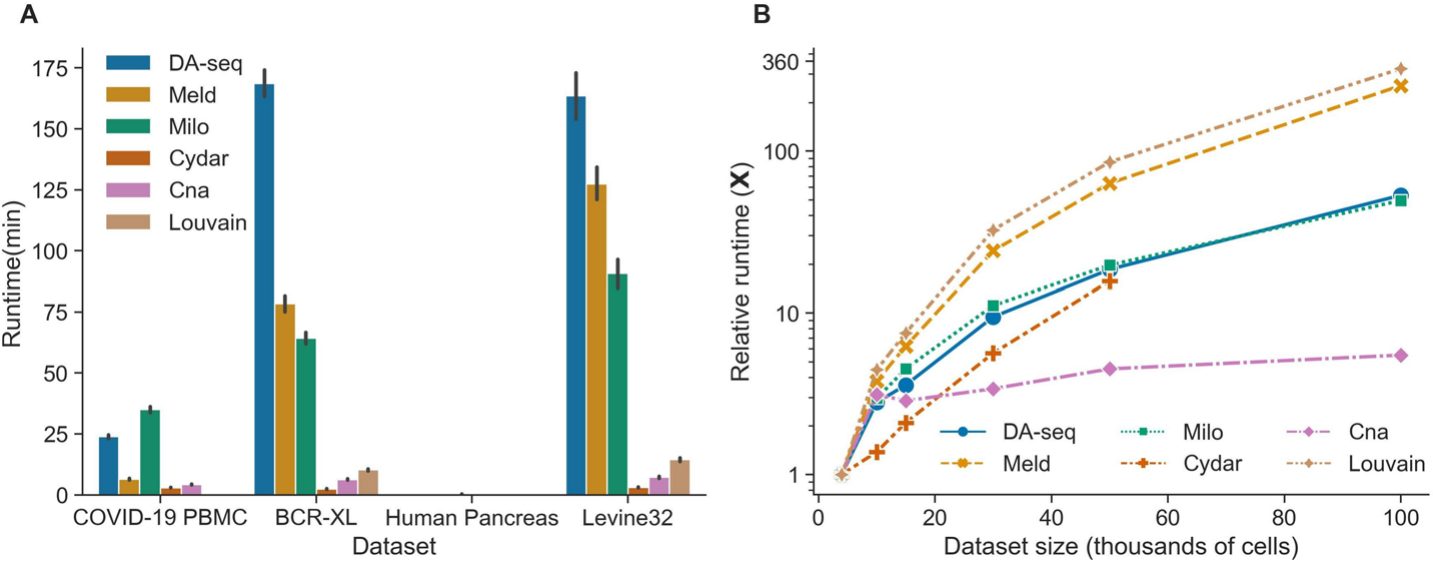
Runtime efficiency and scalability of the six DA testing methods. **A** Runtime of the six DA testing methods on the four real single-cell datasets. The error bars reflect the standard deviation of runtime across various target DA cell populations, DA ratios, and random seeds. The execution times were measured on the nodes of a cluster with Intel Xeon E5-2680 v3 CPUs and 256GB RAM. **B** Relative runtime growth ratio of the six DA testing methods on the single-cell datasets as a function of an increasing number of cells (4k, 10k, 15k, 30k, 50k, and 100k). The runtime of the smallest dataset was used to normalize the runtimes of the larger datasets

Next, we evaluated the runtime efficiency and scalability of the six DA testing methods. We measured the execution time of each method on the four real datasets using the tuned hyperparameters. In the CyTOF datasets containing over 200,000 cells, all methods were able to finish running within a few hours (Fig. 6A). Additionally, some of the DA testing approaches with even higher efficiency, such as Cydar, Cna, and Louvain, only took a few minutes. Thus, we proved that efficiency is not a limiting factor for any of the six DA testing methods when applied to the vast majority of single-cell datasets. Noting that wall-clock runtime depends on numerous factors such as algorithm complexity, hyperparameters, and computing infrastructure, it cannot objectively and completely reflect the scalability of the DA testing methods. As a result, we conducted an additional experiment to quantify the scalability of the DA testing techniques by evaluating the relative runtime growth rate as the number of cells increased. We simulated six single-cell datasets with increasing numbers of cells (4k, 10k, 15k, 30k, 50k, and 100k) using the splatter [18] package. To specifically evaluate the runtimes of the core components of the DA methods without the variable times required for hyperparameter selection, we used default hyperparameters for all datasets. We quantified how well each method scaled by calculating the relative runtime growth with respect to the runtime on the smallest dataset with 4k cells for various data sizes. Figure 6B shows the relative runtime growth as a function of the number of cells in each dataset. Among the six DA testing methods, we discovered that Cna was the most scalable, while Louvain and Meld were the least scalable. The scalability of DA-seq, Milo, and Cydar fell between Cna and Meld, with Cydar being marginally superior to Milo and DA-seq. Notably, the majority of the runtime of the six approaches was spent either counting cells across conditions in cell neighborhoods or building the cell-to-cell graph across samples.

### Hyperparameter tuning and sensitivity

We further evaluated the hyperparameter tunability and sensitivity of the six DA testing methods. Hyperparameters are crucial to the performance of machine learning methods and the best way to identify the optimal hyperparameters is through a line or grid search in hyperparameter space, which takes a lot of time and computational resources. In general, machine learning models with fewer hyperparameters are easier to tune. Furthermore, if a machine learning model’s performance is sensitive to its hyperparameters, it is challenging to identify the best hyperparameters, hence making the model’s performance unstable. Thus, here we examined two major criteria impacting the hyperparameter tuning, including the number of hyperparameters and the overall sensitivity of hyperparameters. The number of hyperparameters reflects how easily a method can be tuned, and sensitivity of hyperparameters measures how stable the DA testing method is overall. Milo and Cna only have one hyperparameter, *k*, which is the number of *k*-nearest neighbors to use in the graph-representation of the data. Alternatively, DA-seq, Meld, Cydar, and Louvain have more hyperparameters (Additional file 1: Table S5). As a result, the hyperparameters of Milo and Cna are easier to tune than those of DA-seq, Meld, Cydar, and Louvain.

**Fig. 7:**
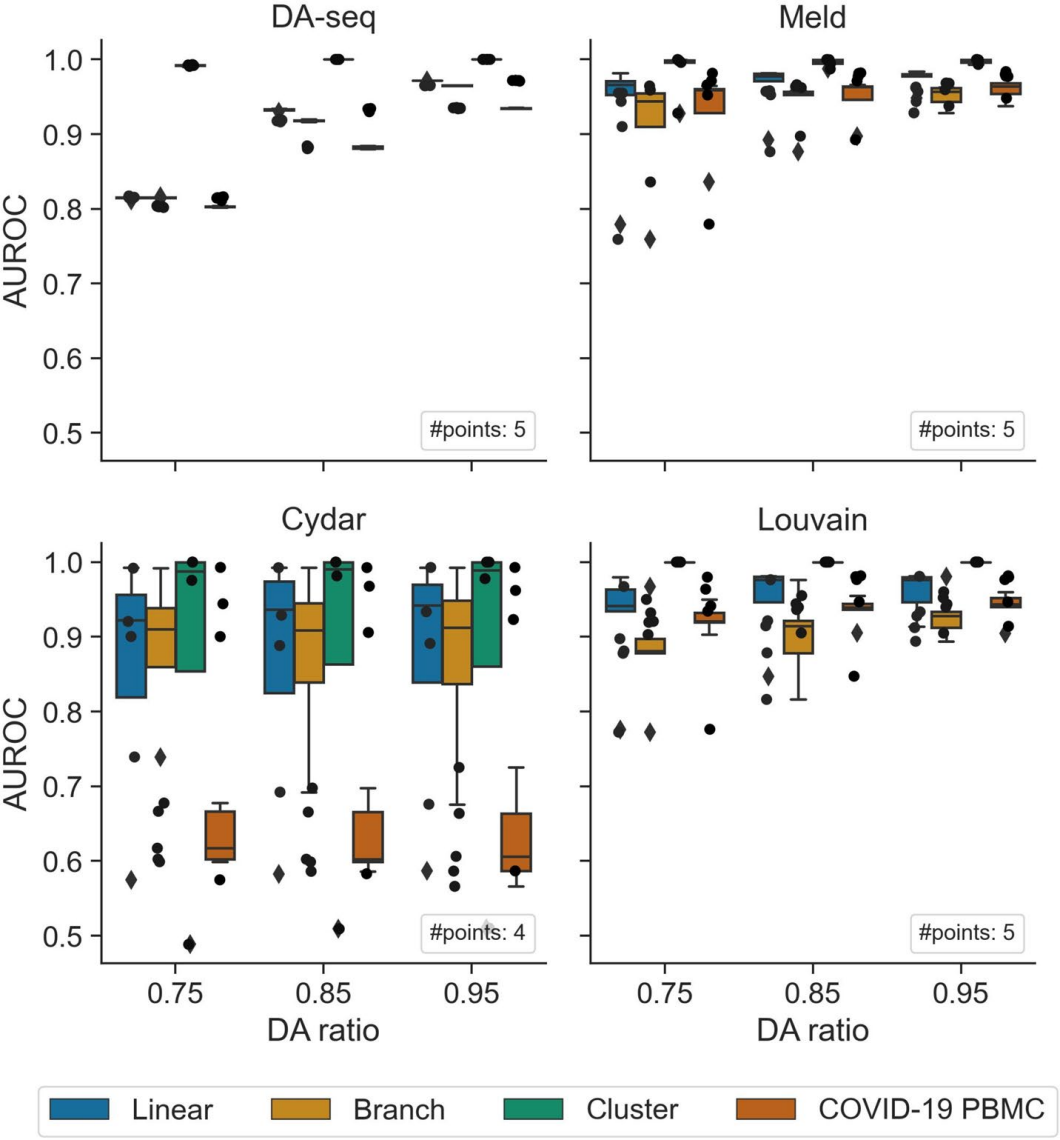
Hyperparameter sensitivity of the six DA testing methods. Performance of the four DA testing methods (DA-seq, Meld, Cydar, and Louvain) on the three synthetic datasets and the COVID-19 scRNA-seq dataset with a range of DA ratios (0.75, 0.85, and 0.95) in the target DA cell population. The boxplots show (e.g., each data point) the distribution of AUROC scores across various hyperparameters. High variance implies sensitivity to choice of hyperparameters. #points represents the number of points for each boxplot

To test the hyperparameter sensitivity of each DA testing method, we evaluated their performance for predicting DA cells on three synthetic datasets (linear, branch, and cluster) and on the COVID-19 PBMC scRNA-seq dataset by altering their hyperparameters. Since Meld, Milo, Cna, and Louvain all share a common hyperparameter, *k*, we fixed *k* and solely tested the hyperparameter sensitivity relative to the other parameters in order to control variable. In contrast to other methods, DA-seq employs a range of hyperparameters $\textbf{k} = [k_1, \ldots , k_l]$ to generate *k*-nearest neighbor graphs. We altered the hyperparameters of DA-seq by replacing $k_1$ with the same *k* used in the other methods, while varying the step size between $k_i$ and $k_{i+1}$. The boxplots in Fig. 7 visualize the variation in performance of DA-seq, Meld, Cydar, and Louvain, with each dot representing a run with specific hyperparameters. First, DA-seq had the lowest overall hyperparameter sensitivity, indicating that users do not need to modify its hyperparameters excessively for practical applications. Second, although having somewhat higher variance than DA-seq, Meld’s performance did not show significant variance. Cydar and Louvain, on the contrary, consistently had high variance in their performances across all datasets. These experiments suggested that Cydar and Louvain are hyperparameter-sensitive. The rationale for Cydar’s high hyperparameter sensitivity is that finding an appropriate radius in high-dimensional space is inherently difficult, as data points become sparser as the dimensionality increases [19]. Louvain’s strong hyperparameter sensitivity is primarily due to the resolution parameter, which ultimately controls the number of clusters identified. Taken together, it is crucial to identify the optimal hyperparameters for Cydar and Louvain for real-world application to ensure their robust performances across all settings.

## Discussion

In this work, we evaluated and compared six prominent single-cell DA testing methods for resolving cell populations in response to external variables, such as clinical phenotype or experimental perturbation. Our benchmarking workflow was designed to cover as many realistic applications of DA testing scenarios as possible, including diverse single-cell data types, various data topologies, and the existence of technique-induced biases. In our experiments, we assessed the DA testing methods using both synthetic and real single-cell datasets with distinct topological structures. In addition, simulated batch effects were generated and applied to the datasets to assess the robustness of DA testing methodologies. Thus, our benchmarking strategies offered a thorough, quantitative evaluation of the DA testing methods. We evaluated the performance of each method by calculating AUROC and AUPRC scores to quantify the similarity between predicted and established ground-truth DA labels. By objectively comparing the performance of the six DA testing approaches on a variety of tasks, we determined that no single method outperformed the others across the board. In other words, the appropriate selection of DA testing methods depends on properties of the data and ultimate task of interest (e.g., the existence of batch effects). In the discussion that follows, we summarize our experimental findings for each given task.

The majority of the DA testing methods examined in our work, particularly Meld, Milo, and Cydar, demonstrated consistently strong accuracy across datasets and DA ratios for identifying DA cell types in synthetic datasets (Fig. 3 and Additional file 1: Tables S3 and S4). Meld performed the best across all approaches in the single-cell datasets, but Milo also attained satisfactory accuracy. When additional technical challenges, such as batch effects were present in the datasets, Milo was the most effective at correcting them and reducing their negative impacts on testing accuracy. In addition, we demonstrated that including batch labels in a DA testing model enhanced performance in comparison to not including them. As for runtime efficiency and overall scalability, all methods can successfully complete their workflows on typically sized scRNA-seq and large CyTOF datasets using standard CPUs in a few hours. Finally, we looked at the sensitivity and usability of the hyperparameters used in the DA testing methods. While Milo required tuning of the fewest hyperparameters, DA-seq and Meld were robust to the selection of hyperparameters. Furthermore, our benchmarking evaluations identified a common problem across the majority of DA testing methods. In particular, all methods performed poorly, even on simple datasets, when a substantial imbalance of cells existed between cell-types. Our hypothesis is that such behavior is caused by local DA heterogeneity in a large cell population. As this issue is seemingly complex, we leave a more in-depth analysis of this phenomenon across methods and datasets to our future work.

## Conclusion

Based on our thorough benchmarking analyses, the following are our general suggestions for the usage of DA testing methods in practice (Additional file 1: Table S7). First, we observed that Meld is the most accurate methods overall when there is no substantial technical noise, such as batch effects. Moreover, in the event of technical or biological noise, Milo performs better on average than Meld. Our experiments further suggested that Milo, Cydar, Cna, and Louvain are all viable candidates for robustly identifying DA cell-populations, while controlling the false discovery rate. Milo, DA-seq, and Meld either have the fewest hyperparameters or are insensitive to hyperparameter changes. Therefore, they are the robust strategies for performing DA testing on a new dataset. Lastly, for large single-cell datasets with many cells, we found Cna to be the most scalable method. The presented benchmarking study has hopefully revealed some critical insights to help users in selecting the optimal differential abundance approach for their specific datasets.

## Methods

### Datasets and data pre-processing

#### Synthetic datasets

For the comparison of DA testing performance, we generated raw count matrices for each of the three synthetic single-cell datasets with different topological structures, including linear trajectories, branching trajectories, and discrete clusters using the generate_dataset() function in the R package dyntoy [20]. Each dataset included six samples from a simulated experiment with three replicates (R1, R2, and R3) and two experimental conditions (C1 and C2). Each dataset varies in their number of total cells. For example, the linear and branch datasets contain 7500 cells and 500 genes, whereas the cluster dataset contains 2700 cells and 500 genes. For the comparison of runtime efficiency and scalability, we simulated six single-cell datasets with increasing numbers of cells (4k, 10k, 15k, 30k, 50k, and 100k) using the splatSimulate() function in the splatter [18] package.

#### Real datasets

##### COVID-19 PBMC Dataset

The COVID-19 PBMC dataset is a scRNA-seq dataset generated by profiling 44,721 peripheral blood mononuclear cells (PBMCs) from seven hospitalized COVID-19 patients, four of whom had acute respiratory distress syndrome, and six healthy controls [7]. Considering that one of the patients was sampled twice, this dataset contains 8 samples with COVID-19 symptoms and 6 healthy controls. In addition to basic clinical outcomes, this dataset contains information regarding the COVID-19 illness trajectory of each patient, such as, severity classification at the time of admission (ICU/Floor), ventilation status, etc. In the original study (Ref. [7]), the authors investigated the changes in cell type proportions between the COVID-19 samples and the healthy controls and revealed that case severity was associated with the depletion or expansion of several canonical immune cell-types, including developing neutrophils and plasmablasts. Therefore, the intended use of this dataset was to examine the efficacy of different DA testing approaches for identifying differentially abundant cell-populations.

##### Human Pancreas Dataset

The Human Pancreas dataset is a scRNA-seq dataset, where samples were collected from pancreatic tissue and cultured islets of six healthy and four Type 2 diabetes donors [16]. For our benchmarking purposes, we exclusively used cells from the healthy donors, which resulted in a dataset of 1,597 cells. This dataset is predominantly comprised of five distinct endocrine cell types: $\alpha$ cells (secreting glucagon, GCG), $\beta$ cells (insulin, INS), $\gamma$/PP cells (pancreatic polypeptide, PPY), $\delta$ cells (somatostatin, SST), and $\epsilon$ cells (ghrelin, GHRL).

##### BCR-XL CyTOF Dataset

The BCR-XL dataset presented in Ref. [14] is comprised of 172,791 human PBMCs collected across 16 CyTOF samples, eight of which were stimulated with B cell receptor/Fc receptor cross-linker (BCR-XL) with the remaining being case controls. Cells in this dataset belong to one of eight manually-gated cell populations, including B-cells IgM-, B-cells IgM+, CD8+ T-cells, CD4+ T-cells, DC, surface-cells, NK cells, and monocytes.

##### Levine32 CyTOF Dataset

This is a CyTOF dataset originally introduced in Ref [15], containing 265,627 healthy human bone marrow mononuclear cells (BMMCs) sourced from two healthy individuals. In this dataset, 14 distinct cell populations were manually gated based on 19 out of the 32 measured surface protein markers.

##### Data Pre-processing

For all the scRNA-seq datasets, we first normalized the raw count matrices using a log+1 transformation. Then, we projected the normalized gene expression data into principal component (PC) space and embedded the data into the manifold approximation and projection (UMAP) [21] space using the top-50 PCs. In our analysis of the COVID-19 PBMC dataset, we directly utilized the previously processed data introduced in Ref. [7], whose processing procedures adhered closely to best practices for the analysis of single-cell data [22]. In addition to the data, the authors also provided embeddings for each cell according to both PCA and UMAP. The PC embeddings were generated using the normalized data of the highly variable genes and the UMAP embeddings were constructed using the top 50 PCs. In all the CyTOF datasets, we normalized all protein expressions with an arcsinh transformation with a cofactor of 5, as suggested in Ref. [23].

##### Generation of ground-truth DA labels

For all the synthetic and real datasets, we generated ground-truth DA labels as follows. First, we selected one of the cell populations as the target population to exhibit differential abundance between the two experimental conditions (conditions denoted by C1 and C2) , and further considered the remaining cells as background. Next, following the same pipeline introduced in Ref. [5], we assigned a given cell, *i*, represented as $\textbf{x}_i$, a probability $P(C2)_i$ of being generated under condition C2 as,

1 $$\begin{aligned} P(C2)_i = \frac{w_i-\textrm{min} \left( w_i\right) }{ \textrm{max} \left( w_i\right) -\textrm{min} \left( w_i\right) }(p_{\textrm{DA}} - 0.5)+0.5. \end{aligned}$$

Here, $w_i$ represents the logit-transformed weighted distance between a cell $\textbf{x}_i$ and the centroid of the target cell population. A cell $\textbf{x}_i$ that is closer to the centroid of the target cell population will result in a higher value of $w_i$, thereby leading to a larger value of $P(C2)_i$. The parameter $p_{\mathrm{DA}}$ denotes the maximal enrichment of condition C2 within the target cell population, thereby illustrating the magnitude of the differential abundance effect, or DA ratio. Given equation (1), a $P(C2)_i$ value of 0.5 indicates no differential abundance, while a value greater than 0.5 suggests a higher abundance of the cell in condition C2. Lastly, we assigned the ground-truth DA label $o_i$ to each cell based on the simulated probability:

2 $$\begin{aligned} o_i{} & {} = \text {NegLFC } (\text {enriched in } \textrm{C} 1), \text { if } P(\textrm{C} 2)_i < t \nonumber \\ o_i{} & {} = \text {PosLFC } (\text {enriched in } \textrm{C} 2), \text { if } P(\textrm{C} 2)_i > 1 - t \nonumber \\ o_i{} & {} = \text {NotDA}, \text {Otherwise}. \end{aligned}$$

Here, the threshold *t* is calculated based on the proportion of the target cell population within the entirety of the dataset. Visual depictions for each synthetic dataset can be found in Additional file 1: Fig. S1.

##### Simulation of batch effects

To simulate batch effects, we first randomly assigned cells across samples to two simulated batches (B1 and B2). Subsequently, a Gaussian random vector was sampled and integrated (added) into the principal component (PC) profile of every cell within each batch. To simulate varying intensities of batch effects, we adjusted the standard deviation (denoted by batchSd) of the Gaussian vector in 0.25 increments, ranging from 0.75 to 1.5.

### Differential abundance (DA) testing methods

#### Problem formulation

We define a sample $\textbf{X}_{n \times p} = (\textbf{x}_1, \ldots , \textbf{x}_n)^\top$ to be the normalized gene or protein expression matrix with *n* cells and *p* measured features, where $\textbf{x}_i = (x_{1i}, \ldots , x_{pi})^\top$ is the feature vector for cell *i*. Given a collection of *N* samples $\{\textbf{X}^k\}_{k=1}^N$ profiled from *N* individuals (donors), with a particular sample *i* associated with a clinical or experimental label $y_i$, the goal of DA testing is to identify a subset of cells exhibiting differential abundance (density) in response to the labels encoded (e.g., using the $y_{i}$s for each individual *i*) across the samples. This DA testing problem can alternatively be stated as a density estimation problem [19]. In this case, each experimental condition can be viewed as a primary distribution, and the objective of DA testing is to detect cells with relatively lower or higher densities under each label or condition. In this subsection, we describe all of the benchmarking methods in this study. For a more detailed introduction about the DA testing methods, please refer to their respective original papers [1, 3–6, 17].

#### Cydar

Cydar [1] is a statistical testing approach developed to identify cell populations in single-cell mass cytometry datasets with a differential abundance of particular cell-types between conditions. Cydar’s central idea is to construct hyperspheres in the multi-dimensional marker space as local units to test if the number of cells among samples in each hypersphere is related to external labels, such as clinical or experimental outcomes. Given an *N*-sample single-cell dataset measuring *p* markers in each cell, Cydar’s testing pipeline works as follows: (1) Cydar randomly samples a subset of cells from the entire dataset and uses these cells as the centers of hyperspheres to allocate cells from all samples to the hyperspheres; (2) Cydar then counts the number of cells assigned to each hypersphere in each sample, resulting in an *N*-dimensional abundance vector; (3) next, Cydar employs the negative binomial generalized linear models (NB-GLMs) in the edgeR package [24] to perform statistical testing on these count data with respect to clinical outcomes and other informational covariates and assigns a *P*-value to each hypersphere; (4) lastly, Cydar identifies the statistically significant hyperspheres as DA regions by controlling the spatial false discovery rate (FDR), a weighted form of FDR that regulates FDR across volume, at a predetermined threshold $\alpha$. Here, Cydar applies the Benjamini-Hochberg (B-H) procedure [25] to calculate the maximum *P*-value needed to keep a hypersphere below the spatial FDR threshold $\alpha$, which is defined as,

3 $$\begin{aligned} \underset{i}{\textrm{max}}\left\{ p_{(i)}: p_{(i)} \le \alpha \frac{\sum _{l=1}^{i} w_{(l)}}{\sum _{l=1}^{n} w_{(l)}}\right\} . \end{aligned}$$

Here, *n* is the number of hyperspheres, $p_{(1)}<p_{(2)}<\ldots <p_{(n)}$ provides an ordering of the *P*-values of the hyperspheres and $w_{(l)}$ is defined as the weight of hypersphere *l*, which is the reciprocal of the density of hypersphere *l*. In our benchmark, Cydar v1.18 (http://bioconductor.org/packages/cydar) was applied across all the experiments.

#### DA-seq

In DA-seq [3], a logistic regression classifier is used to compute a local DA score for each cell so that DA subpopulations can be identified. The logistic regression classifier creates feature vectors for each cell, which reflect the abundance of two biological conditions in the area around each cell at different scales. Using the labels of the samples from which the cells originated, DA-seq trains a logistic regression model. The fitted probability is then used as the DA score for each cell. In this case, the trained logistic regression model serves as a smoothing function that transforms a cell’s input feature vector to its corresponding soft DA score. Next, DA-seq uses a random permutation test to find statistically significant DA cells in the dataset. The upper and lower cut-off thresholds are based on the highest and lowest DA scores inferred under the null hypothesis that the condition labels are distributed randomly. In our experiments, we used the official DA-seq implementation, which can be accessed at https://github.com/KlugerLab/DAseq.

#### Meld

Meld [4] is a graph-based kernel density estimation method. It is used to estimate the likelihood of a sample (in this case, a cell) under various experimental perturbations. Inspired by the recent success of applying manifold learning techniques to single-cell data visualization [21, 26, 27], Meld extends kernel density estimation (KDE) from the regular spatial domain to a manifold represented by a cell-by-cell similarity graph denoted by $\mathcal {G}=(V, E)$. Here, Meld requires two steps to obtain the edge weights in $\mathcal {G}$. First, the Euclidean distance between cells is calculated for a pair of cells, (*i*, *j*). Next, the weight (similarity) between a cell pair (*i*, *j*) by feeding their distance to some predefined kernel functions, such as the $\alpha$-decaying kernel [27] or the MNN kernel [28].

The Meld algorithm interprets the cell label as a signal across the cell-cell similarity network. It employs a low-pass graph filter [29] to denoise the node labels across the graph and uses the smoothed label as the DA score measurement for each cell. Noting that this graph filtering step is performed independently on each condition, the smoothed condition labels for each cell must be normalized (summed to 1) in order to derive the conditional label associated likelihood. For experiments with several experimental and control replicates, the Meld algorithm must be applied to each replicate separately, and the DA scores therefore must be averaged across replicates. Meld uses a heuristic strategy to choose DA cell subpopulations by setting a threshold on the per-cell likelihoods to determine whether a cell is in a zone where a certain label is more or less abundant. We used the Meld python package, which can be accessed at https://github.com/KrishnaswamyLab/MELD.

#### Cna

Cna, or “co-varying neighborhood analysis,” identifies phenotype-associated cell populations by examining cell neighborhoods that co-vary in abundance with respect to certain sample covariates, such as experimental treatment or clinical outcome. Similar to Meld, the Cna approach begins by constructing a *k*-nearest neighbor graph of cells across all samples. Cna adopts the scanpy.pp.neighborhood() function from the scanpy package to encode the neighborhood associations between cells into a sparse weighted adjacency matrix $\textbf{A}$. Next, Cna uses a random walk to calculate the likelihood that the $m\prime$-th cell is in the neighborhood of the *m*-th cell. Formally, this is given by

4 $$\begin{aligned} \textbf{P}_{m \prime \rightarrow m}^{s}:=\left( \textbf{e}^{m \prime }\right) ^\top \tilde{\textbf{A}}^{s} \textbf{e}^{m}. \end{aligned}$$

Here, *s* represents the steps of random walk, $\textbf{e}^m$ and $\textbf{e}^{m\prime }$ are the indicator vector defined at indices *m* and $m\prime$, respectively, and $\tilde{\textbf{A}}$ is the random-walk Markov matrix with self-loops, whose entries are computed as,

5 $$\begin{aligned} \tilde{\textbf{A}}_{m\prime ,m} := \frac{(\textbf{I} + \textbf{A})_{m\prime ,m}}{1 + \Sigma _{m \prime \prime } \textbf{A}_{\varvec{\cdot }, m \prime \prime }}. \end{aligned}$$

Here, $\textbf{I}$ is an identity matrix and $\textbf{A}$ is the weighted adjacency matrix that is computed in the graph building step. Letting *c*(*n*) denote the cells from sample *n*, then $\textbf{R}_{n, m}$ is the expected number of cells that would arrive at the neighborhood of the *m*-th cell after *s* steps of random walking beginning from sample *n*. Formally, this is calculated via $\textbf{R}_{n, m}=\sum _{m \prime \in c(n)} \textbf{P}_{m \prime \rightarrow m}^{s}$. Cna further defines the neighborhood abundance matrix (NAM) $\textbf{Q} \in \mathbb {R}^{n\times m}$ by normalizing the rows of $\textbf{R}$ (summed to 1), where

6 $$\begin{aligned} \textbf{Q}_{n, m}=\frac{\textbf{R}_{n, m}}{\Sigma _{m} \textbf{R}_{n, m}}. \end{aligned}$$

Once the NAM is defined, Cna tests its association with a known sample-level covariate $\textbf{y}$ using a linear regression model. The linear model is formally defined as,

7 $$\begin{aligned} \textbf{y} = \textbf{U}^k \varvec{\beta }^k + \epsilon . \end{aligned}$$

Here, $\textbf{U}^k$ represents the first *k* columns of $\textbf{Q}$’s left matrix of singular vectors $\textbf{U}$, $\varvec{\beta }^k$ is the vector of coefficients, and $\epsilon$ denotes zero-mean Gaussian noise. Thus, the *P*-value is calculated using a multivariate *F*-test for a range of *k*s, such that the one attaining the smallest *P*-value is ultimately selected. To identify the differentially abundant neighborhoods, Cna computes a “smoothed correlation” between each neighborhood *m* and the sample-level covariate $\textbf{y}$. The smoothed correlation is mathematically defined as,

8 $$\begin{aligned} \varvec{\gamma } := \textbf{V}^{k^\star } \textbf{D}^{k^\star } \varvec{\beta }^{k^\star }. \end{aligned}$$

Here, $k^\star$ denotes the optimal number of singular vectors (e.g., components) determined by the multivariate *F*-test, $\textbf{V}^{k^\star }$ is the first $k^\star$ columns of $\textbf{Q}$’s right singular vector matrix, $\textbf{D}^{k^\star }$ is the top-left $k^\star \times k^\star$ submatrix of $\textbf{Q}$’s singular vector matrix, and $\varvec{\beta }^{k^\star }$ is the coefficient vector defined in (7). To assess the statistical significance, the null distribution of $\varvec{\gamma }$ is obtained by fitting (7) using different permutations of $\textbf{y}$. Lastly, the DA cell sub-populations are determined by a given FDR threshold for $\varvec{\gamma }$. The Cna approach is implemented in python and is available at https://github.com/immunogenomics/cna.

#### Milo

As an improved version of Cydar, Milo also uses NB-GLMs to test DA cells in single-cell datasets but replaces the hypersphere in Cydar with cell neighborhoods from the cell-cell similarity graph. Here, the neighborhood of a cell $c_i$ is defined as the set of first order neighbors (including $c_i$ itself) in a *k*NN graph created by the findKNN() function in the BiocNeighbors package. After counting the number of cells in the neighborhoods of several samples, Milo employs the same statistical testing pipeline with edgeR as Cydar, except that the testing unit is a cell-neighborhood instead of a hypersphere. To reduce complexity, Milo samples only a small proportion (by default, 0.1) of cell neighbors to find DA neighborhoods. As various cell neighborhoods may share certain cells in the *k*NN graph, it is vital to highlight that a cell neighborhood must propagate its DA score to each of its respective cells. Hence, the DA score of a tested cell is ultimately determined by adding the DA scores of all the cell neighborhoods to which it belongs. In this work, we used the R-based implementation of Milo (https://github.com/MarioniLab/miloR), as suggested by the authors.

#### Louvain

The Louvain algorithm [17] is a cluster-based approach for DA testing. Unlike other more granular approaches, which are performed on single cells [3, 4], hyperspheres [1], and cell neighborhoods [5, 6], Louvain’s results are typically coarser, operating on a cluster level, and hence can only determine whether a cell cluster is a DA region or not. In other words, if a cell cluster is determined to be a DA cluster, all cells insides this cluster become classified as DA cells with identical DA scores. The Louvain method is implemented as follows: (1) a *k*NN graph or cell-to-cell similarity graph is constructed, (2) the Louvain algorithm partitions the graph into clusters [17] (implementation provided by the cluster_louvain() function in the R package igraph [30]), and (3) apply the statistical framework of Milo [5] to identify DA cell-populations. Note that the Louvain approach does not implement DA-score aggregation step introduced by Milo and therefore produces solely non-overlapping cell clusters.

### Evaluation and metrics

Evaluating and comparing the performance of the various DA testing methods is non-trivial due to their variable testing procedures for identifying and quantifying the significance of DA cells. Cydar, Milo, and Cna, for instance, employ traditional statistical testing measures like FDR and spatial FDR to detect DA cell populations, whereas DA-seq and Meld use a conditional probability threshold. Therefore, it is impossible to develop a uniform criterion that can be consistently applied to all methods. To eliminate the bias of selecting a distinct threshold for each approach, each method’s predicted labels were generated using a range of thresholds based on its own criterion. To quantify overall classification performance, we compared predicted labels with ground truth labels that were generated through simulation for each cell and had three distinct categories: (1) enriched in C1 (NegLFC, negative log fold-change in condition C2 vs. C1), (2) enriched in C2 (PosLFC, positive log fold-change in condition C2 vs. condition C1), and (3) not DA, respectively.

To generate a list of evaluation thresholds, we first calculated DA scores (FDR or conditional probability, depending on the approach) under each method. Next, for each method, we specified the thresholds using the values at different percentiles (by default: 0% to 100% with 1% increments) of its DA scores. We then used the false positive rate (FPR) and true positive rate (TPR), two binary classification metrics, to assess the performance of each approach for each threshold, yielding a list of FPR and TPR pairs. We treated both the PosLFC and NegLFC as the “positive” label of binary classification to account for the fact that there are three possible ground-truth labels. The FPR and TPR are defined respectively as,

9 $$\begin{aligned} \textrm{FPR}{} & {} = \frac{\textrm{FP}}{\textrm{FP} + \textrm{TN}}, \end{aligned}$$

10 $$\begin{aligned} \textrm{TPR}{} & {} = \frac{\textrm{TP}}{\textrm{TP} + \textrm{FN}}. \end{aligned}$$

Here, $\textrm{FP}$ is the number of cells with false positive DA predictions, $\textrm{TN}$ is the number of cells with true negative predictions, $\textrm{TP}$ is the the number of cells with true positive predictions, and $\textrm{FN}$ is the number of cells with false negative predictions. Finally, we connected the 2d-points of the FPR and TPR pairs sequentially with FPR plotted on the horizontal axis and TPR plotted on the vertical axis to construct receiver operator curves (ROC) and reported the area under ROC (AUROC) score as the overall performance for each method. Similarly, we also calculated precision score and used the area under precision-recall (PR) curve (AUPRC) as another complementary metric.

### Supplementary information

**Additional file 1:** **Figure S1.** Two-dimensional UMAP visualization of the three synthetic single-cell datasets, where the cells are colored by target DA cell populations (cell type). **Figure S2.** Two-dimensional UMAP visualization of the four real single-cell datasets, where the cells are colored by their annotated cell types. **Figure S3.** Performance of the six DA testing methods for DA prediction on the synthetic cluster dataset (A) and the *balanced* cluster dataset (B) with a range of DA ratios (0.75, 0.85, and 0.95) in the target DA cell populations (M1, M2, and M3). The boxplots represent the AUROCscores over different random seeds. **Figure S4.** Performance of the six DA testing methods for DA prediction on the synthetic cluster dataset (A) and the *balanced* cluster dataset (B) with a range of DA ratios (0.75, 0.85, and 0.95) in the target DA cell populations (M1, M2, and M3). The boxplots represent the AUPRC scores over different random seeds. **Figure S5.** Performance comparison of DA testing methods by modeling or not modeling batch effects. Performance of the four DA testing methods (Cydar, Milo, Cna, and Louvain) for DA prediction on the three synthetic datasets (linear (left), branch (middle), and cluster (right)) with batch effects of varying magnitudes (from 0 to 1.5), where the colors (orange and blue) indicate whether batch labels are utilized in the model. The boxplots represent the AUROC scores for different target DA cell populations, DA ratios, and random seeds. **Table S1.** The single-cell DA testing methods benchmarked in this study and their basic characteristics. **Table S2.** Hyperparameters of the six single-cell DA testing methods used on the single-cell datasets in the benchmarking experiments. **Table S3.** The corresponding median AUROC scores in Figure 3 for the six DA testing methods on the three synthetic datasets (linear, branch, and cluster) with a range of DA ratios (0.75, 0.85, and 0.95). **Table S4.** The corresponding median AUPRC scores in Figure 3 for the six DA testing methods on the three synthetic datasets (linear, branch, and cluster) with a range of DA ratios (0.75, 0.85, and 0.95). **Table S5.** The corresponding median AUROC scores in Figure 4A for the six DA testing methods on the four real single-cell datasets with a range of DA ratios (0.75, 0.85, and 0.95). **Table S6.** The corresponding median AUPRC scores in Figure 4B for the six DA testing methods on the four real single-cell datasets with a range of DA ratios (0.75, 0.85, and 0.95). Table S7. Overall suggestions for the usage of DA testing methods.

**Additional file 2.** Review history.

## Data Availability

The raw data for the COVID-19 PBMC scRNA-seq dataset is publicly available through the NCBI Gene Expression Omnibus (GEO; https://www.ncbi.nlm.nih.gov/geo/) repository, with accession number GSE150728 [31]. The authors of the original work also provide processed count matrices with manually annotated metadata and pre-computed embeddings in .h5ad and .rds formats, which can be downloaded from the Wellcome Sanger Institute’s COVID-19 Cell Atlas at https://www.covid19cellatlas.org/. The raw data of the Human Pancreas scRNA-seq data is publicly available at the European Nucleotide Archive (ENA; https://www.ebi.ac.uk/arrayexpress/experiments/) repository, under accession number: E-MTAB-5061 [32]. The raw data of the BCR-XL dataset including FCS files and their corresponding metadata and annotations can be downloaded from FlowRepository with accession number FR-FCM-ZYL8 [33]. The Levine32 CyTOF dataset is available in FlowRepository with accession number FR-FCM-ZZPH [34]. All code to reproduce results are available at https://github.com/CompCy-lab/benchmarkDA and in the Zenodo repository [35]. Source code is released under the MIT license.
