# Supplementary material for: Benchmarking differential abundance methods for finding condition-specific prototypical cells in multi-sample single-cell datasets
Source: Genome Biol. 2024 Jan 3;25:9. doi: 10.1186/s13059-023-03143-0 (PMC10762948; doi:10.1186/s13059-023-03143-0)
Supplement: Supplementary file 1 — Additional file 1: Figure S1. Two-dimensional UMAP visualization of the three synthetic single-cell datasets, where the cells are colored by target DA cell populations (cell type). Figure S2. Two-dimensional UMAP visualization of the four real single-cell datasets, where the cells are colored by their annotated cell types. Figure S3. Performance of the six DA testing methods for DA prediction on the synthetic cluster dataset (A) and the balanced cluster dataset (B) with a range of DA ratios (0.75, 0.85, and 0.95) in the target DA cell populations (M1, M2, and M3). The boxplots represent the AUROCscores over different random seeds. Figure S4. Performance of the six DA testing methods for DA prediction on the synthetic cluster dataset (A) and the balanced cluster dataset (B) with a range of DA ratios (0.75, 0.85, and 0.95) in the target DA cell populations (M1, M2, and M3). The boxplots represent the AUPRC scores over different random seeds. Figure S5. Performance comparison of DA testing methods by modeling or not modeling batch effects. Performance of the four DA testing methods (Cydar, Milo, Cna, and Louvain) for DA prediction on the three synthetic datasets (linear (left), branch (middle), and cluster (right)) with batch effects of varying magnitudes (from 0 to 1.5), where the colors (orange and blue) indicate whether batch labels are utilized in the model. The boxplots represent the AUROC scores for different target DA cell populations, DA ratios, and random seeds. Table S1. The single-cell DA testing methods benchmarked in this study and their basic characteristics. Table S2. Hyperparameters of the six single-cell DA testing methods used on the single-cell datasets in the benchmarking experiments. Table S3. The corresponding median AUROC scores in Figure 3 for the six DA testing methods on the three synthetic datasets (linear, branch, and cluster) with a range of DA ratios (0.75, 0.85, and 0.95). Table S4. The corresponding median AUPRC scores in Figure 3 for th [file 13059_2023_3143_MOESM1_ESM.pdf]

Benchmarking differential abundance methods for finding condition-specific prototypical cells in multi-sample single-cell datasets: supplementary figures and tables

HAIDONG YI, ALEC PLOTKIN, NATALIE STANLEY

1. SUPPLEMENTARY FIGURES

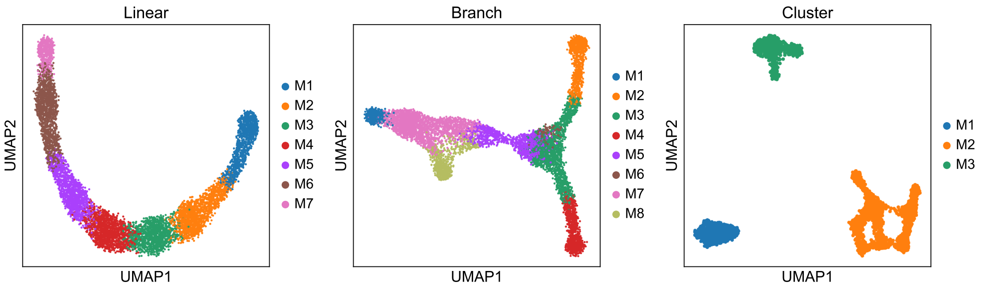

Figure S1. Two-dimensional UMAP visualization of the three synthetic single-cell datasets, where the cells are colored by target DA cell populations (cell type).

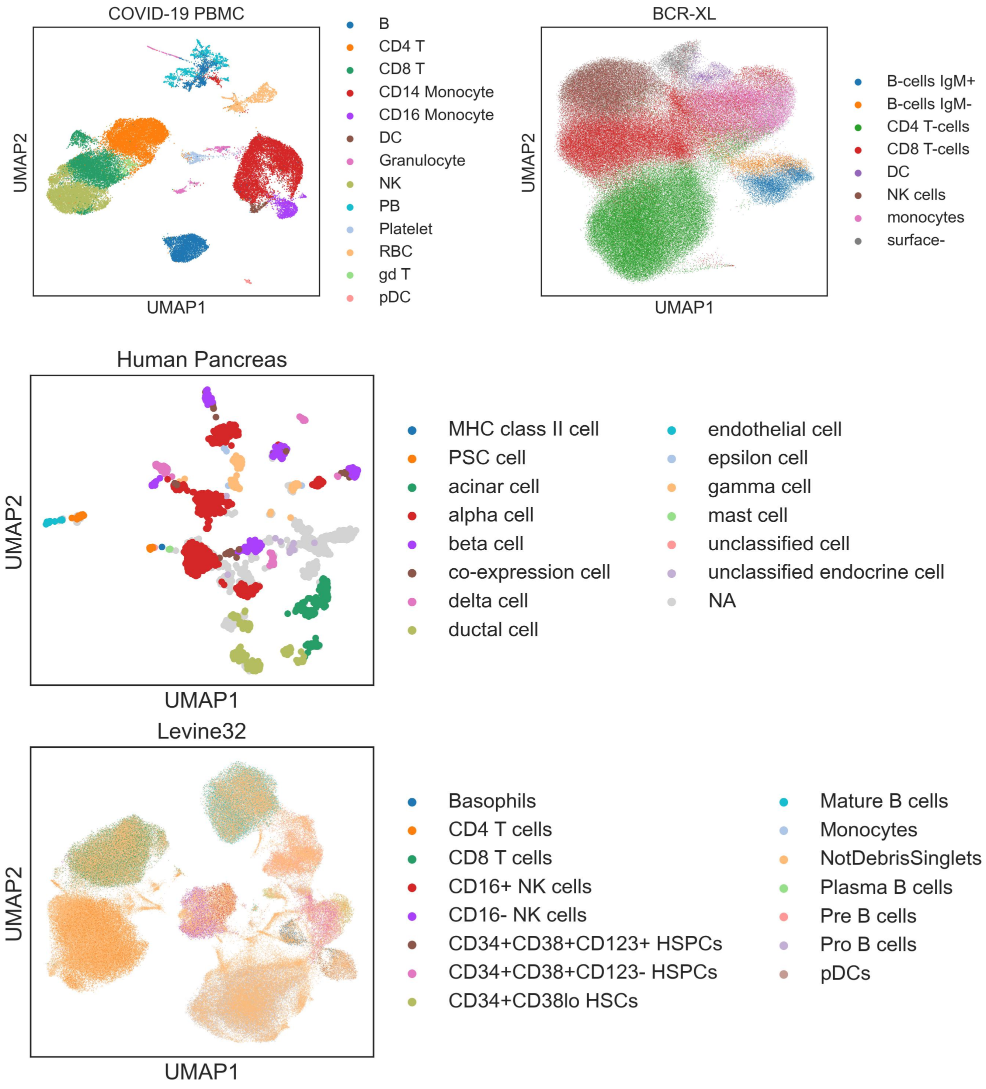

Figure S2. Two-dimensional UMAP visualization of the four real single-cell datasets, where the cells are colored by their annotated cell types.

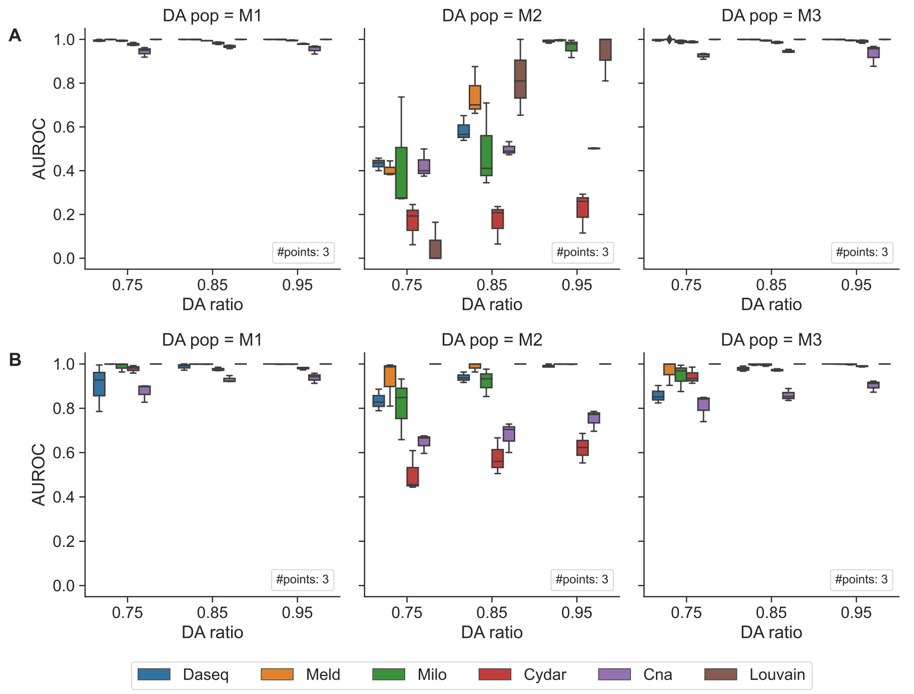

Figure S3. Performance of the six DA testing methods for DA prediction on the synthetic cluster dataset (A) and the *balanced* cluster dataset (B) with a range of DA ratios (0.75, 0.85, and 0.95) in the target DA cell populations (M1, M2, and M3). The boxplots represent the AUROC scores over different random seeds.

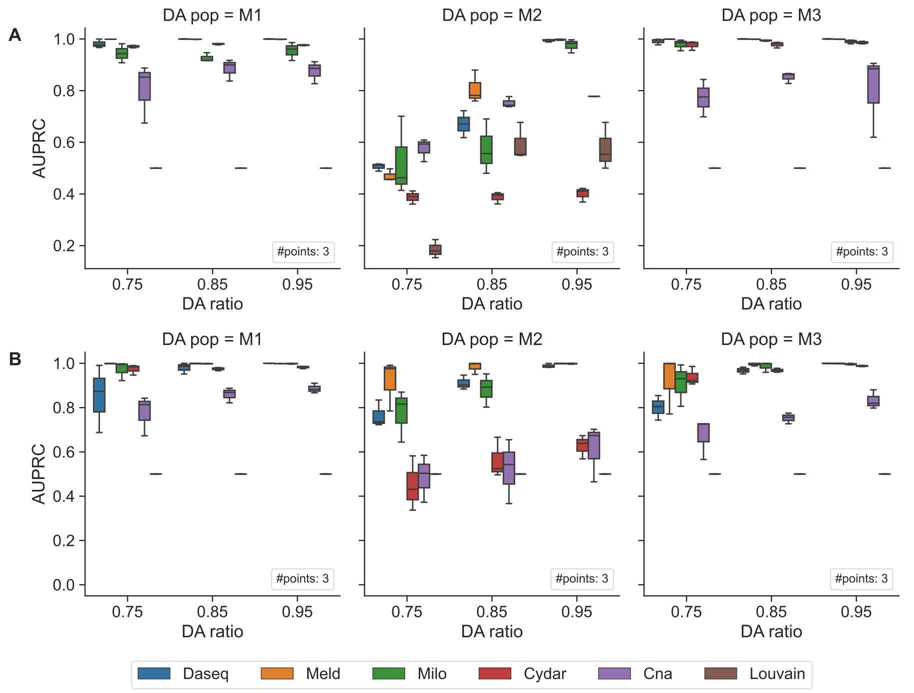

Figure S4. Performance of the six DA testing methods for DA prediction on the synthetic cluster dataset (A) and the *balanced* cluster dataset (B) with a range of DA ratios (0.75, 0.85, and 0.95) in the target DA cell populations (M1, M2, and M3). The boxplots represent the AUPRC scores over different random seeds.

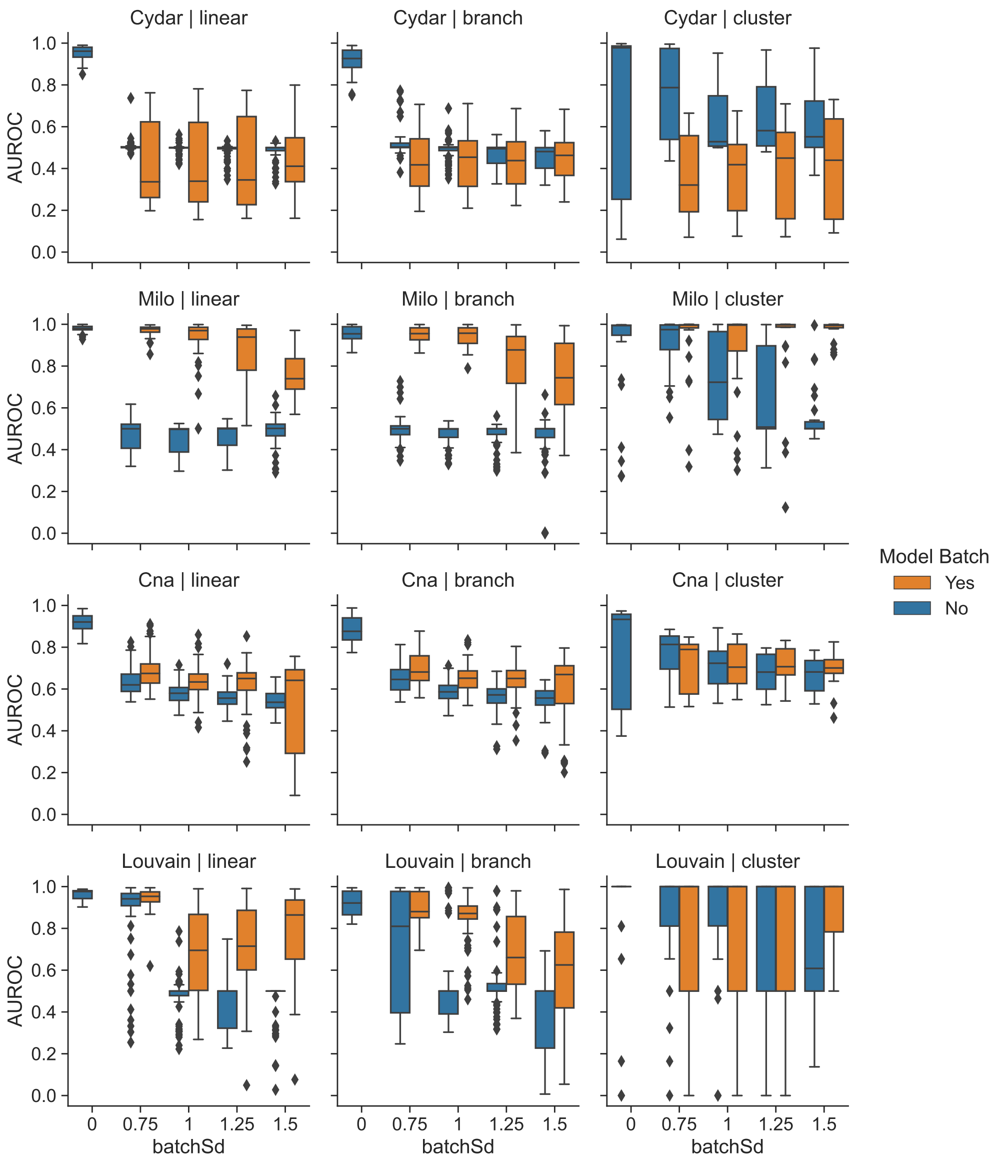

Figure S5. Performance comparison of DA testing methods by modeling or not modeling batch effects. Performance of the four DA testing methods (Cydar, Milo, Cna, and Louvain) for DA prediction on the three synthetic datasets (linear (left), branch (middle), and cluster (right)) with batch effects of varying magnitudes (from 0 to 1.5), where the colors (orange and blue) indicate whether batch labels are utilized in the model. The boxplots represent the AUROC scores for different target DA cell populations, DA ratios, and random seeds.

2. SUPPLEMENTARY TABLES

Table S1. The single-cell DA testing methods benchmarked in this study and their basic characteristics.

Methods	Handle experimental covariates	Statistical Testing	Approach	DA cells selection	Language
Cydar [1]	✔	✔	NB-GLM of Hyperspere	False Discovery Rate	R
Louvain [2]	✔	✔	NB-GLM of Cluster	False Discovery Rate	R
Milo [3]	✔	✔	NB-GLM of *k*NN Graph	False Discovery Rate	R/Python
Cna [4]	✔	✔	SVD of NAM*	False Discovery Rate	Python
DAseq [5]	✗	✔	Logistic Regression	DA score	R (depends on Python)
Meld [6]	✗	✗	Graph Density Estimation	Likelihood	Python

*: NAM represents the neighborhood abundance matrix.

Table S2. Hyperparameters of the six single-cell DA testing methods used on the single-cell datasets in the benchmarking experiments. Here, k represents the number of neighborhoods used to build the kNN graphs, $\mathbf{k} = [k_1, \ldots, k_l]$ is a range of k parameters used to build kNN graphs in DA-seq [5], β is a hyperparameter that controls the smoothness of graph density estimation in Meld [6], tol is a hyperparameter that determines the hypersphere radius used in Cydar [1], and res is the resolution parameter used in the Louvain clustering [2].

Methods	linear	branch	cluster	COVID-19 PBMC	BCR-XL	Human Pancreas	Levine32
DA-seq [5]	$\mathbf{k} = [30, \ldots, 480]$, where $k_{l+1} - k_l = 50$.						
Meld [6]	k : 30, β : 71	k : 30, β : 65	k : 30, β : 33	k : 30, β : 25	k : 30, β : 23	k : 30, β : 80	k : 30, β : 36
Milo [3]	k : 30						
Cydar [1]	tol : 2.3	tol : 2.4	tol : 2.8	tol : 2.1	tol : 0.75	tol : 2.85	tol : 0.45
Cna [4]	k : 30						
Louvain [2]	k : 30, res : 1	k : 30, res : 1	k : 30, res : 0.2	k : 30, res : 0.5	k : 30, res : 0.6	k : 30, res : 1.2	k : 30, res : 0.6

Table S3. The corresponding median AUROC scores in Figure 3 for the six DA testing methods on the three synthetic datasets (linear, branch, and cluster) with a range of DA ratios (0.75, 0.85, and 0.95).

	branch			cluster			linear		
	0.75	0.85	0.95	0.75	0.85	0.95	0.75	0.85	0.95
DA-seq [5]	0.815	0.917	**0.965**	0.992	0.999	0.999	0.816	0.934	0.972
Meld [6]	0.955	0.956	0.963	**1.000**	**1.000**	**1.000**	**0.981**	0.982	0.984
Milo [3]	0.932	**0.962**	0.971	0.991	0.994	0.995	0.974	**0.982**	**0.985**
Cydar [1]	0.921	0.929	0.934	0.976	0.982	0.978	0.944	0.968	0.962
Cna [4]	0.856	0.884	0.880	0.942	0.933	0.933	0.911	0.929	0.938
Louvain [2]	0.878	0.921	0.928	1.000	1.000	1.000	0.964	0.977	0.977

Table S4. The corresponding median AUPRC scores in Figure 3 for the six DA testing methods on the three synthetic datasets (linear, branch, and cluster) with a range of DA ratios (0.75, 0.85, and 0.95).

	branch			cluster			linear		
	0.75	0.85	0.95	0.75	0.85	0.95	0.75	0.85	0.95
DA-seq [5]	0.519	0.769	0.898	0.977	1.000	1.000	0.543	0.810	0.913
Meld [6]	0.814	0.860	0.880	0.999	0.998	0.999	0.917	0.909	0.920
Milo [3]	0.769	0.834	0.866	0.944	0.917	0.983	0.897	0.920	0.920
Cydar [1]	0.779	0.785	0.0.794	0.966	0.977	0.976	0.849	0.884	0.900
Cna [4]	0.502	0.565	0.617	0.699	0.837	0.827	0.583	0.702	0.711
Louvain [2]	0.436	0.456	0.442	0.500	0.500	0.500	0.466	0.476	0.466

Table S5. The corresponding median AUROC scores in Figure 4A for the six DA testing methods on the four real single-cell datasets with a range of DA ratios (0.75, 0.85, and 0.95).

	BCR-XL			COVID-19 PBMC			Human Pancreas			Levine32		
DA ratio	0.75	0.85	0.95	0.75	0.85	0.95	0.75	0.85	0.95	0.75	0.85	0.95
DA-seq	0.774	0.896	0.957	0.803	0.882	0.935	0.694	0.853	0.970	0.802	0.916	0.971
Meld	0.983	0.984	0.983	0.959	0.966	0.968	0.712	0.859	0.916	0.982	0.978	0.965
Milo	0.904	0.948	0.963	0.840	0.902	0.930	0.627	0.771	0.881	0.962	0.980	0.984
Cydar	0.702	0.754	0.758	0.602	0.602	0.606	0.698	0.842	0.913	0.985	0.985	0.986
Cna	0.922	0.952	0.961	0.821	0.869	0.904	0.661	0.794	0.905	0.834	0.909	0.941
Louvain	0.943	0.945	0.944	0.921	0.944	0.944	0.620	0.772	0.875	0.961	0.961	0.961

Table S6. The corresponding median AUPRC scores in Figure 4B for the six DA testing methods on the four real single-cell datasets with a range of DA ratios (0.75, 0.85, and 0.95).

dataset	BCR-XL			COVID-19 PBMC			Human Pancreas			Levine32		
DA ratio	0.75	0.85	0.95	0.75	0.85	0.95	0.75	0.85	0.95	0.75	0.85	0.95
DA-seq	0.312	0.499	0.710	0.351	0.567	0.702	0.249	0.509	0.804	0.309	0.528	0.701
Meld	0.792	0.808	0.798	0.738	0.807	0.835	0.231	0.461	0.551	0.722	0.737	0.732
Milo	0.538	0.631	0.696	0.529	0.685	0.722	0.202	0.359	0.651	0.548	0.682	0.744
Cydar	0.298	0.331	0.315	0.306	0.345	0.371	0.083	0.317	0.659	0.740	0.742	0.684
Cna	0.469	0.576	0.619	0.310	0.480	0.582	0.165	0.366	0.442	0.271	0.386	0.470
Louvain	0.309	0.316	0.308	0.398	0.417	0.393	0.068	0.134	0.312	0.219	0.219	0.219

Table S7. Overall suggestions for the usage of DA testing methods.

Case	Methods					
	DA-seq[5]	Meld[6]	Milo[3]	Cydar[1]	Cna[4]	Louvain[2]
w/o technical noise (e.g. batch effects)	✔	✔	✔	✔	✗	✗
w/ technical noise (e.g. batch effects)	✗	✗	✔	✗	✗	✗
w/ Type I error control	✗	✗	✔	✔	✔	✔
robust to hyperparameter	✔	✔	✔	✗	✗	✗
scalable to data size	✗	✗	✗	✗	✔	✗

REFERENCES

1. A. T. Lun, A. C. Richard, and J. C. Marioni, “Testing for differential abundance in mass cytometry data,” Nat. Methods **14**, 707–709 (2017).
2. V. D. Blondel, J.-L. Guillaume, R. Lambiotte, and E. Lefebvre, “Fast unfolding of communities in large networks,” J. statistical mechanics: theory experiment **2008**, P10008 (2008).
3. E. Dann, N. C. Henderson, S. A. Teichmann, M. D. Morgan, and J. C. Marioni, “Differential abundance testing on single-cell data using k-nearest neighbor graphs,” Nat. Biotechnol. **40**, 245–253 (2022).
4. Y. A. Reshef, L. Rumker, J. B. Kang, A. Nathan, I. Korsunsky, S. Asgari, M. B. Murray, D. Moody, and S. Raychaudhuri, “Co-varying neighborhood analysis identifies cell populations associated with phenotypes of interest from single-cell transcriptomics,” Nat. Biotechnol. **40**, 355–363 (2022).
5. J. Zhao, A. Jaffe, H. Li, O. Lindenbaum, E. Sefik, R. Jackson, X. Cheng, R. A. Flavell, and Y. Kluger, “Detection of differentially abundant cell subpopulations in scrna-seq data,” Proc. Natl. Acad. Sci. **118**, e2100293118 (2021).
6. D. B. Burkhardt, J. S. Stanley, A. Tong, A. L. Perdigoto, S. A. Gigante, K. C. Herold, G. Wolf, A. J. Giraldez, D. van Dijk, and S. Krishnaswamy, “Quantifying the effect of experimental perturbations at single-cell resolution,” Nat. Biotechnol. **39**, 619–629 (2021).
