# Supplementary material for: Benchmarking differential abundance methods for finding condition-specific prototypical cells in multi-sample single-cell datasets
Source: Genome Biol. 2024 Jan 3;25:9. doi: 10.1186/s13059-023-03143-0 (PMC10762948; doi:10.1186/s13059-023-03143-0)
Supplement: Supplementary file 2 — Additional file 2. Review history. [file 13059_2023_3143_MOESM2_ESM.docx]

**1at round**

**Reviewer 1**

Yi et al. present a benchmarking study of computational methods for identifying differentially abundant cell populations in single cell data, including both single-cell transcriptomics and CyTOF data. The study evaluated a total of six methods using simulated data and two publicly available single-cell datasets. The work is timely, and the authors have chosen representative of the state-of the-art methods for differential abundance analysis. The conclusions drawn from the study are interesting and can help potential users select the method best suited to their needs. Crucially, the authors have made the code for the analyses presented in the manuscript available. This is important, as it will allow researchers developing new methods to benchmark them using the same pipeline.

In my opinion, there are two important areas in which the study could be improved:

1. The manuscript should provide more detail about the methodology used to benchmark the methods. This information is critical for readers to fully understand the scope and significance of the results presented. Specifically:

1a. The authors do not present detailed information about how the ground-truth labels were constructed in both the simulated and real datasets, as well as the number of labels for each class in each dataset.

1b. Including details about the specific parameters used in the simulations, as well as the number of simulations of each type, would also substantially improve the clarity of the manuscript. Additionally, it should be clarified whether the authors used dyntoy, Splatter, or both to produce the synthetic datasets, as this is currently unclear from the text. For example, whereas in subsection "Synthetic Datasets" it is stated that the synthetic datasets were produced using dyntoy, in subsection "Benchmarking overview" it is stated that they used both dyntoy and Splatter.

1c. To aid in the interpretation of the results, specifying the number of points in each boxplot in each boxplot in the legends for the figures would be beneficial.


2. There are some limitations to the extent of the evaluations that need to be addressed:

2a. The overall performance of each method was evaluated using the AUC score, which may be misleading when data is imbalanced. To provide a more comprehensive analysis the authors could consider presenting the area under the precision-recall curve (AUC-PR) in addition to the AUC.

2b. The evaluation is limited by the number of real datasets with known ground-truth that were used (n = 2). To improve the evaluation, additional datasets could be constructed by sampling cells from each cell population from other publicly available datasets, where each condition would correspond to a different choice for the sampling probabilities for some cell populations. These datasets would help to complement the purely synthetic data and the two complete single-cell datasets currently used in the manuscript. By including a more diverse set of datasets, the evaluation would provide a more representative and reliable assessment of the methods.


In addition, I observed several typos:

- Second line of the "Background" section: "genes of tens" -> "genes or tens"
- Sixth line of subsection "DA testing performance on synthetic datasets": "50 principle components" -> "50 principal components"
- First paragraph in page 8: "Figure 1A" -> "Figure 4B"
- Paragraph after equation (3) of the Methods section: "markov matrix" -> "Markov matrix"

**Reviewer 2**

SUMMARY

The authors present a study benchmarking six methods for differential abundance (DA) analysis on single-cell data.
Using simulated and two real datasets, the authors assess these methods in terms of area under the ROC curve, robustness to and/or treatment of batch effects, and running time.
In their discussion, the author recommend DA methods based attributes of the data being analyzed (mainly: presence of batch effects), and based other considerations (e.g., robustness, scalability).

GENERAL COMMENTS

Differential abundance analyses of single-cell data address questions that arise naturally in many application contexts (e.g., healthy vs. disease samples), and a thorough and systematic comparison of available methods would be useful for the community of scientists analyzing these data. However, there are some substantial concerns I have with the manuscript in its current form:

- The relationship between differential expression and differential abundance analysis should be explicitly discussed in the introduction, and connections to similar setups (e.g. microbiome) should be mentioned and put into context.

- With only two real datasets analyzed (one scRNA-seq, one cytof), it is not clear if observations will generalize to other data. The rationale for studying these two datasets should be made clear. More real data would strengthen the conclusions.

- The manuscript is not overly concise, however key information is not provided. For example, detailed explicit description and motivation for how the synthetic data was generated is missing, making it hard to evaluate results; but also see specific comments below.

- Code and data should be provided for reproducibility; providing the data used is also on of the key elements that makes benchmark studies useful for the community.

- It is unclear wy AUC (and only AUC) was chosen as a performance metric; other measures (mcc, average precision, f1-score) should also be considered. Further on, it would be useful to test if performance is significantly different between some of the methods, using some reasonable hypothesis testing setup.

The authors' result that Meld and Milo perform well on their data seems reasonably well supported and is useful, especially if it holds for more than two datasets.
The point about all methods performing poorly with imbalanced cell types appears to be made with synthetic data entirely and it not entirely clear (e.g., in figure S2 some gains in the M2 population in the balanced setup appears to trigger worse performance for M1 and M3); the clarity of this result could be improved.


SPECIFIC COMMENTS

- In results frequently AUC values reported are much smaller than 0.5, which is what is expected under random guessing. It is unclear how to interpret this: Is this noise? If not, what should we interpret these observations?

- A more detailed explanation would make figure 1 more clear

- There is some repetition in describing the data used in the methods and in the results section.

- It is unclear how the synthetic data is generated.

- Ref 14 has also assessed performance of Cydar, DAseq, Louvain (and Milo). Do your assessments agree with theirs? What are key differences or similarities?

- The term "DA-ratio" is not defined. Why the the range for DA-ratio between .75 and .95?

- It is not clear how "ground truth" labels were defined for the two real datasets

- In the simulated model, how precisely are batch effects included, and what is batchSD (how does its magnitude relate to a effect size/signal)?

- In Figure 5 the colors for Milo and Cydar appear swapped

**Authors’ response**

Reviewer 1

**Yi et al. present a benchmarking study of computational methods for identifying differentially abundant cell populations in single cell data, including both single-cell transcriptomics and CyTOF data. The study evaluated a total of six methods using simulated data and two publicly available single-cell datasets. The work is timely, and the authors have chosen representative of the state-of the-art methods for differential abundance analysis. The conclusions drawn from the study are interesting and can help potential users select the method best suited to their needs. Crucially, the authors have made the code for the analyses presented in the manuscript available. This is important, as it will allow researchers developing new methods to benchmark them using the same pipeline.**

**In my opinion, there are two important areas in which the study could be improved: *Major comments:***

**Q1: The manuscript should provide more detail about the methodology used to bench mark the methods. This information is critical for readers to fully understand the scope and significance of the results presented. Specifically:**

**Q1a: The authors do not present detailed information about how the ground-truth labels were constructed in both the simulated and real datasets, as well as the number of labels for each class in each dataset.**

A1a: We appreciate the constructive feedback. In our methodology, we generated the ground truth differential abundance (DA) labels for each cell, according to the method used by *Dann, Emma, et al., 2022*. To further clarify how these ground-truth DA labels were created, we have incorporated a detailed explanation of this approach in the "Methods-Generation of ground-truth DA labels" section. It is worth noting that real single-cell datasets (e.g. non-synthetic) do not inherently have ground-truth labels, or a notion of which cells are differentially abundant. Hence, for the real datasets, we adopted the same approach as with synthetic ones, but leverage cell populations with validated cell type labels as target populations to simulate differential abundance with respect to.

**Q1b: Including details about the specific parameters used in the simulations, as well as the number of simulations of each type, would also substantially improve the clarity of the manuscript. Additionally, it should be clarified whether the authors used dyntoy, Splatter, or both to produce the synthetic datasets, as this is currently unclear from the text. For example, whereas in subsection "Synthetic Datasets" it is stated that the synthetic datasets were produced using dyntoy, in subsection "Benchmarking overview" it is stated that they used both dyntoy and Splatter.**

A1b: Thank you for pointing this out. We have included the specific use of hyperparameters across our experiments in Supplementary Table S2. In our simulations, we employed both the dyntoy and Splatter but for different uses. We used the dyntoy to generate diverse synthetic datasets to compare the performance of DA testing methods (see updated explanation in the

first sentence of "Methods–Synthetic Datasets". Because dyntoy is practically quite slow in simulating large single-cell datasets, we used splatter to evaluate scalability (see Updated explanation of this in the last sentence of "Methods-Synthetic Datasets").

**Q1c: To aid in the interpretation of the results, specifying the number of points in each boxplot in each boxplot in the legends for the figures would be beneficial.**

A1c: The number of points has now been included in the legends for each figure containing boxplots.

**Q2: There are some limitations to the extent of the evaluations that need to be addressed:**

**Q2a: The overall performance of each method was evaluated using the AUC score, which may be misleading when data is imbalanced. To provide a more comprehensive analysis the authors could consider presenting the area under the precision-recall curve (AUC-PR) in addition to the AUC.**

A2a: Thank you for this excellent comment. We have now included the Area Under the Precision Recall Curve (AUPRC) in the performance evaluation for all experiments. Note that we introduced this metric in the "Methods-Evaluation and Metrics" section of all of our experiments, reflected in Figures 3 and 4.

**Q2b: The evaluation is limited by the number of real datasets with known ground truth that were used (n 2). To improve the evaluation, additional datasets could be constructed by sampling cells from each cell population from other publicly available datasets, where each condition would correspond to a different choice for the sampling probabilities for some cell populations. These datasets would help to complement the purely synthetic data and the two complete single-cell datasets currently used in the manuscript. By including a more diverse set of datasets, the evaluation would provide a more representative and reliable assessment of the methods.**

A2b: Thank you for the comments. To obtain more reliable and representative assessment of the methods, we introduced two additional real single-cell datasets, including *Human Pancreas (* Segerstolpe, Åsa, et al., 2016*) (single-cell RNA Sequencing)* and *Levine32 (*Levine, Jacob H., et al., 2015*), CyTOF*. Note that descriptions of these datasets have been added to sections "Methods-Levine32 CyTOF Dataset" and "Methods-Human Pancrease Dataset". In our revised manuscript, we have updated all experiments to include these two datasets, impacting figures 4 and 6, and supplementary table S5&S6. We showed that these two additional datasets exhibited similar behavior as the other real single-cell datasets.

***Minor comments:***

**Q1: Second line of the "Background" section: "genes of tens" -> "genes or tens"** A1: Thank you, we have made this change.

**Q2: Sixth line of subsection "DA testing performance on synthetic datasets": "50 principle components" -> "50 principal components"**

A2: Thank you for catching this, we have made this change.

**Q3: First paragraph in page 8: "Figure 1A" -> "Figure 4B"**

A3: Thank you, we have made this change.

**Q4: Paragraph after equation (3) of the Methods section: "markov matrix" -> "Markov matrix"**

A4: Thank you, we have made this change.

Reviewer 2

**The authors present a study benchmarking six methods for differential abundance (DA) analysis on single-cell data. Using simulated and two real datasets, the authors assess these methods in terms of area under the ROC curve, robustness to and/or treatment of batch effects, and running time. In their discussion, the author recommend DA methods based attributes of the data being analyzed (mainly: presence of batch effects), and based other considerations (e.g., robustness, scalability).**

***General comments:***

**Differential abundance analyses of single-cell data address questions that arise natu rally in many application contexts (e.g., healthy vs. disease samples), and a thorough and systematic comparison of available methods would be useful for the community of scientists analyzing these data. However, there are some substantial concerns I have with the manuscript in its current form:**

**Q1: The relationship between differential expression and differential abundance analysis should be explicitly discussed in the introduction, and connections to similar setups (e.g. microbiome) should be mentioned and put into context.**

A1: Thank you giving us the opportunity to clarify the nuances of these classes of approaches. We added a discussion about these points, as well as about contrasts with approaches in microbiome analysis in the first paragraph of "Background".

**Q2: With only two real datasets analyzed (one scRNA-seq, one cytof), it is not clear if observations will generalize to other data. The rationale for studying these two datasets should be made clear. More real data would strengthen the conclusions.**

A2: Thank you for this excellent point, which is in line with the comments of Reviewer 1. In response, we added two additional real single-cell datasets, including *Human Pancreas (* Segerstolpe, Åsa, et al., 2016*) (single-cell RNA Sequencing)* and *Levine32 (*Levine, Jacob H., et al., 2015*), CyTOF*. Note that descriptions of these datasets have been added to sections "Methods-Levine32 CyTOF Dataset" and "Methods-Human Pancreas Dataset". In our revised manuscript, we have updated all experiments to include these two datasets, impacting figures 4 and 6, and supplementary table S5&S6. We showed that the two additional datasets had similar conclusions as the other datasets across all experiments.

**Q3: The manuscript is not overly concise, however key information is not provided. For example, detailed explicit description and motivation for how the synthetic data was gen erated is missing, making it hard to evaluate results; but also see specific comments below.**

A3: Thank you for pointing this out. In our methodology, we generated the ground-truth differential abundance (DA) labels for each cell, aligning with the pipeline originally described in *Dann, Emma, et al., 2022*. In the revised manuscript, we’ve incorporated a detailed expla nation in section "Methods-Generation of Ground Truth DA Labels". Briefly, in this approach, specific cell types are chosen to be modeled as abundant under a certain condition, with the probability based on a function inversely related to the distance from the population’s centroid.

**Q4: Code and data should be provided for reproducibility; providing the data used is also on of the key elements that makes benchmark studies useful for the community.**

A4: We agree with this comment and have made sure that code to reproduce our results are available in the following public repository, https://github.com/CompCy-lab/benchmarkDA. We added a link to this repository in the “Availability of data and materials” section at the end of the manuscript.

**Q5: It is unclear why AUC (and only AUC) was chosen as a performance metric; other measures (mcc, average precision, f1-score) should also be considered. Further on, it would be useful to test if performance is significantly different between some of the methods, using some reasonable hypothesis testing setup.**

A5: Thank you for pointing this out. In the revised manuscript, we explained the reason for this point in the section “Methods-Evaluation and Metrics”. For reviewers’ convenience, we will also briefly address this point here. Since the measures used in different methods for differential abundance testing are different, it is hard to find a uniform criterion that can be consistently applied to all methods. For example, Milo used FDR to detect DA cell populations, whereas DA-seq and Meld used a conditional probability threshold. For each method, we adhered to its own cut-off thresholds to distinguish between DA and NotDA cells. By calculating the AUC score using FPRs and TPRs at these varying thresholds, we mitigated biases introduced when utilizing disparate measures for DA testing across the methods. To make the comparison more comprehensive, we have incorporated the area under precision-recall (PR) curve (AUPRC) as an ancillary metric in the revised manuscript. For mcc and f1-score, we didn’t adopt it since they can only be calculated at a specific cut-off threshold.

**Q6: The authors’ result that Meld and Milo perform well on their data seems reasonably well supported and is useful, especially if it holds for more than two datasets. The point about all methods performing poorly with imbalanced cell types appears to be made with synthetic data entirely and it not entirely clear (e.g., in figure S2 some gains in the M2 population in the balanced setup appears to trigger worse performance for M1 and M3); the clarity of this result could be improved.**

A6: Thank you for your insightful comments. In the revised manuscript, we have elaborated on the contexts to clarify the reasons behind the observed results (see paragraph 2&3 in section “Results-DA testing performance on synthetic datasets”). All methods consistently underperformed when targeting the M2 DA cell populations. Upon analyzing the predictions across all methodologies, we found more false negative (FN) predictions. This suggests that a segment of cells within the M2 population was misclassified as non-DAs, even though they should be identified as DAs. Given that M2 contains a higher cell count than M1 and M3, we infer that there’s inherent DA heterogeneity within the M2 cell populations. When we balanced the cell counts across M1, M2, and M3, this heterogeneity within M2 was reduced, leading to notably improved performance across all methods.

***Specific comments:***

**Q1: In results frequently AUC values reported are much smaller than 0.5, which is what is expected under random guessing. It is unclear how to interpret this: Is this noise? If not, what should we interpret these observations?**

A1: Thank you for pointing this out. In our implementation, we used the sklearn.metrics.auc() function from the scikit-learn package to compute the area under the curve (AUC). For some cases that AUCs are below 0.5, these can be thought of as random due to the poor

performance. We attribute this to potential factors like simulated batch effects or cell hetero geneity.

**Q2: A more detailed explanation would make figure 1 more clear**

A2: We have updated the figure 1 in the revised manuscript and added more explanations in the caption of figure 1.

**Q3: There is some repetition in describing the data used in the methods and in the results section.**

A3: Thank you for pointing this out. To eliminate the repetition, we have moved all the data descriptions to the section “Methods-Datasets and Data Pre-processing”.

**Q4: It is unclear how the synthetic data is generated.**

A4: Thank you for this constructive comment. We have added more details (e.g. functions used from the packages) about how the synthetic datasets and the ground-truth labels are simulated in the section “Methods-Synthetic Datasets” and “Methods-Generation of ground truth DA labels” in our revised manuscript.

**Q5: Ref 14 has also assessed performance of Cydar, DAseq, Louvain (and Milo). Do your assessments agree with theirs? What are key differences or similarities?**

A5: Thank you for pointing this out. Overall, our results align well with those reported in Ref. 14 (*Dann, Emma, et al., 2022*). For example, both studies identified the Milo method (Ref. 14) as the most effective method among the four compared. Similarly, DA-seq in Ref. 14 was determined to be average in performance (Figure 2 in Ref. 14), a conclusion mirrored in our study (see Figure 3&4). Ref. 14 also highlighted that the Louvain method exhibited higher FDR values, an observation consistent with the poor AUPRC scores in our results for Louvain (see Figure 3&4). These observations reaffirm the consistency of our outcomes with the conclusions drawn in Ref. 14. Notably, while Ref. 14 evaluated performance based on FPRs and FDRs across several thresholds, our benchmarking incorporated AUROC and AUPRC scores, offering a broader and more comprehensive assessment.

**Q6: The term "DA-ratio" is not defined. Why the the range for DA-ratio between .75 and .95?**

A6: The DA-ratio represents the magnitude of the differential abundance effect existing in the single-cell datasets. In our revised manuscript, we have given its mathematical definition and how we used it for generating the ground-truth DA labels in the section“Methods-Generation of ground-truth DA labels”. We used the range between 0.75 and 0.95 because those are common differentially abundant proportions and we also empirically verified them by coloring the probability *P*(*C*2) in the UMAP visualizations.

**Q7: It is not clear how "ground truth" labels were defined for the two real datasets**

A7: Since like the synthetic datasets, the real datasets don’t also don’t have “ground truth” DA labels, we used the same approach as was applied to the synthetic datasets to model whether or not a given cell-type should be modeled as a DA cell-population, based on ground truth cell-type labels provided. That is, particular cell-types of interest were treated as a group of cells that should have a probability of being a DA population. Please see the first para graph of the section “Methods-Generation of ground-truth DA labels” for a further discussion.

**Q8: In the simulated model, how precisely are batch effects included, and what is batchSD (how does its magnitude relate to a effect size/signal)?**

A8: We appreciate this constructive feedback. In revised manuscript, we’ve included an additional paragraph in the method section “Simulation of batch effects”, detailing our ap proach for incorporating batch effects into the synthetic datasets. Initially, cells across samples were randomly assigned to two simulated batches (B1 and B2). Subsequently, a Gaussian random vector was sampled and integrated into the principal component (PC) profile of every cell within each batch. To simulate varying intensities of batch effects, we adjusted the standard deviation (batchSd) of the Gaussian vector in 0.25 increments, ranging from 0.75 to 1.5. Herein, the batchSd serves as an indicator of the batch effect magnitude within the dataset.

**Q9: In Figure 5 the colors for Milo and Cydar appear swapped**

A9: Thank you for catching this point. We have corrected the color mappings.

In conclusion, we greatly appreciate the reviewers’ comments and believe we have addressed all of the general and specific concerns. These comments have greatly improved the quality of our manuscript. Thank you.

**2^nd^ round**

**Reviewer 1**

I would like to thank the authors for their reply. They have addressed all my comments in the manuscript and I do not have further suggestions.

I noticed a typo in the revised version, in line 27 of page 2: "tends" --> "trends", which the authors may want to fix.

**Reviewer 2**

Overall the manuscript has benefitted from the revision and the authors were able to address my concerns. One remaining issue I have with AUROCs being consistenly below 0.5 (e.g., Figure 5 dataset = linear). In these cases inverting the order would significantly improve the AUC; beause these scores appear systematically smaller than 0.5 I've trouble following the rationale of the authors that this should be due to (independent) noise. I'm not sure what could cause this, but the authors should at least discuss this peculiar underperformance in the manuscript.

**Authors’ response**

Reviewer 1

**Q1: I noticed a typo in the revised version, in line 27 of page 2: "tends" --> "trends",**

**which the authors may want to fix.**

A1: Thank you, we have made this change.

Reviewer 2

**Q1: One remaining issue I have with AUROCs being consistently below 0.5 (e.g., Figure 5 dataset linear). In these cases inverting the order would significantly improve the AUC; because these scores appear systematically smaller than 0.5 I’ve trouble following the rationale of the authors that this should be due to (independent) noise. I’m not sure what could cause this, but the authors should at least discuss this peculiar underperformance in the manuscript.**

A1: Thank you for your perceptive comments regarding the AUROCs below 0.5, particularly in Figure 5 with the linear dataset. We appreciate your suggestion about potentially inverting the order to improve AUROC. In response, we would like to clarify that the < 0.5 AUROC values in our study are a result of specific experimental conditions. Firstly, we deliberately added batch effects into the dataset to test the resilience of various methods under realistic and challenging conditions. This intentional design is crucial in understanding the reduced AUROC scores, as these batch effects make the problem more challenging.

Your observation brings to light an interesting aspect of our findings with respect to the differential impact of these conditions on various methods. For instance, methods like Milo, Meld, and DA-seq showed a significant improvement in AUROC when the order was inverted, while others like Louvain and Cydar did not exhibit the same level of adaptability. This suggests a varying sensitivity to batch effects among different methods, which is an important consideration in our analysis. Since we used the same hyperparameters across orders in Figure 5 for each method, the unexpected trend for Cydar and Louvain can also attribute to their high sensitivity to hyperparameter choice, which is consistent to our results in section: “Hyperparameter tuning and sensitivity”.

In addition, the prediction problem we are addressing is unbalanced, a factor known to negatively impact AUROC scores. The imbalance in our dataset likely biases the performance estimation, especially for AUROC, due to skewed classification thresholds. Given your valuable feedback, we have incorporated a more detailed discussion on these aspects in Results-DA testing performance on datasets with additional technical and biological covariates section. These discussions specifically address how batch effects and data imbalance affect the performance of different methods and how these factors contribute to the observed under-performance in AUROC. By doing so, we aim to provide a more comprehensive understanding about how different methods respond to these challenges.
